# Development and validation of nurse’s assessment ability questionnaire in delirium subtypes: Based on Delphi expert consensus

**DOI:** 10.1371/journal.pone.0297063

**Published:** 2024-01-23

**Authors:** Wen Zhou, Qiulan Zheng, Miao Huang, Jiao Wang, Xiuni Gan

**Affiliations:** 1 Department of Nursing, The Second Affiliated Hospital of Chongqing Medical University, Chongqing, China; 2 Second Department of Nursing School, Chongqing Medical University, Chongqing, China; 3 Basic Department of Nursing School, Chongqing Medical University, Chongqing, China; 4 Joint Surgery Department, The Second Affiliated Hospital of Chongqing Medical University, Chongqing, China; Lahore Medical and Dental College, PAKISTAN

## Abstract

**Background:**

Delirium, a common occurrence in clinical work, can be divided into three subtypes according to *Diagnostic and Statistical Manual of Mental Disorders*, *5 th Edition (DSM-5)*. Each subtype has its special significance and focus. As the primary caregivers and observer of delirious patients, nurses should be able to quickly and accurately indentify each subtype. Therefore, it is necessary to clarify nurses’ assessment ability of delirium subtypes. However, there is currently no suitable questionnaire available for investigating nurses’ assessment ability of delirium subtypes.

**Objective:**

To develop a scientifically validated questionnaire for assessing nursing assessment ability of delirium subtypes based on Knowledge-Attitude-Practice(KAP) Model.

**Methods:**

The questionnaire was conducted from October 2021 to February 2022 to assess the KAP status of nurses the regarding delirium subtype. A two-round Delphi Method was employed to revise the draft questionnaire, ensuring the importance and rationality of each item. Ten experts specializing in critically ill patients, clinical nursing, and nursing management were invited from seven provinces in China for the Delphi process. Additionally, we validated the reliability and validity of the questionnaire.

**Results:**

The return rate in the first and second rounds were 83% and 100%, respectively. The individual authority coefficients for the two rounds of correspondence ranged from 0.787 to 0.987, while the overall authority coefficient of experts was 0.866. Kendall’s coefficient of coordination for the importance scores were found to be 0.192 and 0.156, respectively, whereas those for rationality scores were calculated as 0.149 and 0.141, respectively. Notably, all mean values of importance and rationality scores in the two rounds were exceeded a threshold of 4.10 across both rounds of assessment with coefficient variations (CV) ranging from 0.00 to 0.19 for importance ratings and 0.00 to 0.16 for rationality ratings, both of which were <0.25. Experts proposed modifications to eleven items while introducing four new ones into consideration during this process; thus ensuring that reliability and validity standards were met by the final questionnaire design which consists of a total of thirty-seven items distributed across four dimensions: delirium subtype-related knowledge, assessment attitude, assessment practice, and knowledge source–thereby establishing its clinical relevance as a reliable scientific instrument.

**Conclusion:**

The development process is both scientific and theoretical, encompassing reliable expert correspondence results and a diverse range of question formats. As thus, effectively captures the current landscape of delirium subtypes assessment among clinical nurses from multiple perspectives, including knowledge level and source, attitude, assessment behavior, and assessment barriers. It offers comprehensive and detailed insights.

## 1. Introduction

Delirium, Delirium, a complex psychiatric disorder characterized by impaired consciousness, acute onset, fluctuating course, and inattention [1], is a state of transient confusion caused by multiple factors. Studies have shown the common delirious phenomenon in kinds of patients, including intensive care patients, postoperative patients and so on [2]. Delirium maybe lead to poorer outcomes, including increasing risk of re-intubation and transfering, and impacting patient prognosis [3, 4]. According to the *DSM-5* [1], combined with clinical manifestations, delirium can be divided into three subtypes including hypoactive, hyperactive, and mixed [5].

Different delirium subtypes necessitate distinct care priorities and yield diverse clinical outcomes [6–8]. Hyperactive delirious patients exhibit agitated motor symptoms, rendering them highly susceptible to falls; hence, healthcare professionals should allocate increased attention towards preventing adverse events in this population. Conversely, hypoactive delirious patients manifest subdued symptoms that may be easily overlooked by medical staff. When a patient experiences fluctuations between hypoactive and hyperactive symptoms within a 24-hour period, they are diagnosed with mixed delirium.Early identification of high-risk factors specific to different subtype patients followed by targeted treatment is pivotal for the prevention and management of delirium [9, 10].

Nurses play a crucial role in the identification of delirium subtypes as they are often the primary caregivers for patients.Their expertise in delirium knowledge, accurate determination of subtypes, and effective management of patients with each subtype significantly contribute to improving diagnosis, management, and patient prognosis. However, previous systematic reviews have highlighted deficiencies in nurses’ ability to assess delirium subtypes accurately [11]. Sun’s Chinese research also revealed that only 17.6% of hypoactive delirium cases were identified by nurses [12]. Another review reported that two-thirds of patients with hypoactive delirium were missed [13]. These studies collectively emphasize the urgent need for enhancing clinical nurses’ ability to assess delirium subtypes and also suggests that there is still much room for delirium subtypes assessment. However, to our knowledge, there were no suitable measurement to evaluate the subtype assessment ability, hindering scientific and clear expression.

Therefore, our team aimed to develop a reproducible questionnaire for assessing clinical nurses’ ability to identify delirium subtypes in order to address the delirium subtypes research gap. The objective of this study was to create a scientifically sound questionnaire that could clarify nurses’ knowledge, attitudes, and practices (KAP) related to delirium subtype assessment. To achieve this goal, we utilized the Knowledge-Attitude-Practice Model as a theoretical framework for developing the questionnaire draft. This model is widely used in health and nursing research due to its well-established causal link between knowledge, attitude, and practice [14, 15]. As KAP model shown, knowledge is the basis for practice change, which is a necessary but insufficient condition. Knowledge is the foundation, attitude is the motivation, and practice is the goal.

Besides, our plan was to use the KAP questionnaire as an indicator of clinical nurses’ ability regarding delirium subtype assessment and then refine it through expert consensus using the Delphi Method–a qualitative research method commonly employed in health systems evaluation and decision-making processes [16, 17]. The Delphi method is a prediction and evaluation method that combines quantitative and qualitative evaluation of research objects which is widely used in the health system for evaluation, analysis of decision making, and planning of health development. By combining these two approaches, we aimed to produce a more scientifically rigorous questionnaire.

## 2. Method

### 2.1 Setting up a research group

In this study, our research team comprised 10 members, including 8 clinical nursing experts specializing critical care nursing, anesthesia nursing, geriatric nursing, nursing management, nursing education. Additionally, two master’s degree students majoring in nursing were also part of the team. The educational structure of our research group consisted of one member with a doctoral degree, four members with master’s degrees, two members pursuing there master’s degree as undergraduates, and three members holding bachelor’s degrees. The technical structure was characterized as three chief nursing officers, one vice-chief nursing officer, four moderate nurses, and two primary (supervisor) nurses.

After thorough discussion, the research team members unanimously selected the KAP model as the theoretical framework for this study. Subsequently undergraduates, as well as primary (supervisor) nurses, developed the initial questionnaire entries and designed a draft of the Delphi expert consensus questionnaire. Following this, four moderate nurses and two primary (supervisor) nurses engaged in extensive discussions to refine both the consensus questionnaire and KAP questionnaire entries. The first version of the Delphi questionnaire and KAP questionnaire was then meticulously revised by four officers before being edited by two primary nurses. All comments and suggestions received Delphi process were thoroughly deliberated upon and determined by all research members involved.

Throughout the reseach process, three chief nursing officers provided supervision while maintaining regular. Additionnally, one vice-chief nursing officer along with four moderate nurses conducted confirming final version activities as well as formal survey. Furthermore, data collection and analysis tasks were undertaken by two primary nurses who also managed email correspondence and distribution of formal questionnaires.

### 2.2 Designing the first questionnaire draft

The research team members conducted a comprehensive search using the following keywords: "delirium," "subtypes," "quiet delirium," "agitated delirium," "mixed delirium," "assessment," and "assessment tool" across 11 databases, including China Knowledge Network (CNKI), Wanfang, China Medical Literature Database (CBM), Pubmed, Cochrane Library, Web of Science, CINAHL, Embase, PsycINFO, and Scopus. Subsequently, the research team engaged in extensive discussions to categorize and extract relevant information while designing the item expressions. These efforts were guided by multiple guidelines and tailored to align with the current state of delirium assessment within the Chinese clinical environment.The initial version of the questionnaire was developed through a pre-consultation process followed by iterative revisions based on group discussions.

### 2.3 Delphi method design

In this study, we employed a Delphi Consensus method to assess the importance and rationality of each item for developing a questionnaire regarding clinical nurses’ KAP of delirium subtype assessment. The Delphi method is a qualitative research approach that facilitates consensus-building among experts by soliciting their opinions on real-world problems, offering flexibility in its implementation. Typically, two or three rounds are reached among experts [16, 18] One underlying assumption is that “Identified experts can share their subjective ideas and opinions and these can be captured in narratives provided (Round 1 of the Delphi) and modified based on feedback (second and subsequent iterations of the Delphi)” [19, 20]. One of the advantages is that the Delphi method lies in preserving respondent anonymity, thereby minimizing group pressure for conformity [21]. This methodology finds extensive application within nursing research domains such as nursing education, clinical identification practices, formulation of nursing guidelines, and development of questionnaires [22].

### 2.4 Selection of experts

The experts were selected according to purposive sampling method. The inclusion criteria for expert selection included: 1) clinical experts in critical care medicine or critical care nursing, psychiatric nursing, anesthesia nursing, geriatric nursing, and nursing management; 2) working in this field for ≥ 10 years; 3) holding at least moderate technical titles or master’s degrees; 4) voluntary participation and high motivation.

### 2.5 Design of expert consensus questionnaire

The Delphi consensus questionnaire was initially developed for the delirium subtypes KAP questionnaire, which consisted of four parts:1) Questionnaire Guideline, including the introduction of topic, introduction of the researcher and team, and instructions for filling out the form; 2) General information of experts, including gender, age, education, technical title, research field, and work field; 3) Expert rating form, in which experts were asked to rate the importance and rationality of each item separately, using the Likert 5-point scale, from "very important"/"very rational" scoring 5 points to "unimportant"/"unrational" scoring 1 point. The experts should make comments such as additions, modifications, and deletions; 4) the experts’ self-assessment on the basis of judgment and familiarity with the delirium subtype assessment field.

### 2.6 Consensus questionnaire distribution and items revision

From October to December 2021, the researchers distributed the first round consensus questionnaires by emails or *questionnaire stars* (a free and open online survey website platform). The contents of the questionnaires in both forms were identical, which was convenient for the experts choosing. After completing the first round of consultation, the experts’ opinions were summarized, analyzed, and discussed by the research team. The revised results have been shown in the second round consensus questionnaire to be evaluated again. The second round of consultation was conducted from January 2021 to February 2022. The interval between the two rounds of questionnaires was four weeks. The item revision criteria as following: 1) the mean score of importance or rationality was less than 4 points; 2) the coefficient of variation(CV) ≥25%; 3) experts pointed out the revised, additional, or deleted comments.

When two-round Delphi finished, we invited four professionals (three head nurses and one physician) to join our focus group. The focus group members including our research team members and new participants. Each member (four head nurses, one doctor, three specialist nurses, and five graduate students) was asked to make suggestions on the structure of the questionnaire and the readability of each item. They were asked to consider whether the expression of each item was understandable and ambiguous. All members agreed that each item was understandable and that the structure was reasonable. Additionally, we invited 26 clinical nurses to participate in the preliminary survey and required them to make suggestions regarding the expression of each item. The final version of the KAP questionnaire was affirmed following all discussion.

### 2.7 Data analysis and processing

All data were typed into Excel and calculated by SPSS 26.0. All data were statistically processed by double entry into Excel and calculating. According to previous research and Delphi guideline, the return rate was used to measure the positivity of experts which should be at least 50%, and return rate ≥70% indicate a high level of motivation. The authority coefficient (Cr) was used to measure the degree of authority of the experts, and more credible results were shown while Cr≥0.7. The mean score and coefficient of variation (CV) were used to measure the concentration of the importance and rationality. Kendall’s coefficient of coordination (W-values) was used to indicate the degree of consistency of expert opinion. P<0.05 indicates that the difference is statistically significant. The final deletion and optimization were discussed and decided by the research group.

### 2.8 Ethical aspects

The Ethics Committee approved the study of the Second Affiliated Hospital of Chongqing Medical University (Ethical Approval Number: 2022–11). The acceptance of consensus invitation means informed consent and volunteer. Also, we provided the introduction of this survey and informed consent on the first page of the Consensus questionnaire. The research promised autonomy, anonymity, and no harm, according to the Declaration of Helsinki. The survey did not include any patients or animals during the research process.

## 3. Results

### 3.1 Basic information of experts

We invited twelve experts and ten experts from the seven provinces of Chongqing, Guizhou, Hubei, Shandong, Gansu, Jiangxi, and Heilongjiang to participate in this 2-round correspondence. The recall rates in the first round and second round were 83.3% (12 invited, 10 agreed) and 100% (10 invited, 10 agreed), respectively. According to the high prevalence of delirium in the preliminary literature research and combined with the actual situation in China, delirium assessment mainly focused on critical care. In this Delphi research, clinical experts whose occupational fields were critical care, clinical nursing, and nursing management were invited to this study. More information is shown in Table 1.

**Table 1:** Basic information of experts.

Items		Number(Percent)
Gender	Male	1(10%)
	Female	9(90%)
Age	30–39	2(20%)
	40–49	4(40%)
	≥50	4(40%)
Years of work	10–19	2(10%)
	20–29	5(50%)
	≥ 30	3(30%)
Technical Title	Moderate	1(10%)
	Vice Senior	2(20%)
	Chief Senior	7(70%)
Occupational field*	Critical care	9(90%)
	Clinical nursing	4(40%)
	Nursing Management	5(50%)
Teaching Qualifications	Clinical Teaching	3(30%)
	Master’s degree supervisor	4(40%)
	Doctoral supervision	3(30%)

*: One expert maybe have more than one kind of occupational field.

### 3.2 Authority coefficient of experts

The authority coefficient of experts is calculated by the authority of academic level(q), the weight value of experts’ judgment basis to delirium subtypes assessment, and the weight value of experts’ familiarity. The calculation principle is shown in Table 2. Individual expert authority coefficient = Σ N indicators corresponding value/N = (q+Ca+Cr)/3; overall expert authority coefficient = Σ authority coefficient of each expert/number of experts. The individual authority coefficients of experts for the two rounds in this research were 0.787 to 0.987, all of which were > 0.7, shown in Table 3.

**Table 2:** The calculation principle of authority coefficient.

Part 1 the authority of academic level(q): according to experts’ technical title							
Chief senior		Vice senior		Moderate		junior	
1.0		0.8		0.6		0.4	
Part 2 the weight value of judgment basis(Ca)							
		High(weight value = 1.0)		Middle(weight value = 0.8)		Low(weight value = 0.6)	
Theoretical analysis		0.4		0.3		0.2	
Practical experience		0.4		0.3		0.2	
Literature reading		0.1		0.1		0.1	
Intuition feeling		0.1		0.1		0.1	
Part 3 the weight value of familiarity(Cr)							
Extremely familiar	Very familiar		Generally familiar		Slightly familiar		Unfamiliar
1.0	0.8		0.6		0.4		0.2

**Table 3:** Experts’ authority coefficient.

No.	Academic level(q)	Judgment basis(Ca)	Familiarity(Cr)	Total	Individual Authority coefficient
1	1.0	0.8	0.8	2.60	0.867
2	1.0	0.96	1	2.96	0.987
3	1.0	0.62	0.8	2.42	0.807
4	1.0	0.62	0.8	2.42	0.807
5	0.8	0.98	0.8	2.58	0.860
6	0.8	0.94	0.8	2.54	0.847
7	1.0	0.64	0.8	2.44	0.813
8	1.0	0.94	1	2.94	0.980
9	0.6	0.96	0.8	2.36	0.787
10	1.0	0.94	0.8	2.74	0.903

### 3.3 Concentration and coordination of expert opinions

The concentration degree of experts’ opinions was reflected by the mean and CV of the importance and rationality evaluation. In this study, the mean values of importance and rationality scores in the two rounds were >4.10, indicating that experts considered the importance and rationality of each questionnaire entry to be high. In the first round of correspondence, the CVs of importance ratings ranged from 0.00 to 0.28, the CVs of several individual items were >0.25, and the CVs of rationality ratings were 0.00 to 0.15. The CVs of importance ratings and rationality ratings were 0.00 to 0.19 and 0.00 to 0.16, respectively, both of which were <0.25. It was indicated that experts who approved of the importance and rationality of each item tended to be consistent. The degree of approval tends to be consistent. Kendall’s concordance coefficient were 0.192 and 0.156 for the importance scores and 0.149 and 0.141 for the rationality scores (P-value <0.05), respectively. Table 4 has shown more details.

**Table 4:** Concentration and coordination of expert opinions.

		Score	CV	Kendall’s concordance coefficient		
		($\bar{x}$)		W	χ^2^	P
First-round	Importance	4.20~5.00	0.00~0.28	0.192	75.053	0.000
	Rationality	4.10~5.00	0.00~0.16	0.149	58.029	0.025
Second-round	Importance	4.40~5.00	0.00~0.15	0.156	67.071	0.011
	Rationality	4.60~5.00	0.00~0.16	0.141	60.624	0.039

### 3.4 Final questionnaire confirmation after Delphi consensus

After the first round of expert consultation, experts proposed modifications to nine items and added four. After the second round of consultation, experts proposed modifications to two questions, shown in Table 5. The final questionnaire, 37 items in total, was formed by four dimensions: delirium subtype-related knowledge, assessment attitude, assessment Practice, and knowledge source, as shown in Table 6. The knowledge dimension had 11 items, including one true or false question and ten multiple choice questions; the attitude dimension had 16 items, including 10 scales, five multiple choice questions and one single choice question; the practice dimension had 5 items, including four single choice questions and one multiple choice question; the knowledge sources dimension had 5 items, including one scale, one single choice question and three multiple choice questions.

**Table 5:** Expert comments.

Round	Opinion		Whether to adopt
1	Modify	Knowledge 1.4, risk factors for delirium, options reflecting specific factors	Yes
1		Knowledge 1.6 changed the title from "Assessment/diagnostic tools for delirium" to "Assessment tools for delirium."	Yes
1		Knowledge 1.7, add the option "Targeted treatment according to different subtypes of delirium."	Yes
1		Knowledge 1.8, delete the option "Timely subtype assessment of high-risk patients and targeted preventive treatment for different types of delirium."	Yes
		Knowledge 1.9, delete the options "Patients with increased activity delirium are relatively more likely to have adverse events such as falls, bed falls, and catheter extraction accidents," "Decreased activity delirium is less likely to be noticed by health care workers and has a more serious impact on patients."	Yes
		Attitudes 2.13 changed the section to "The nurses’ recognition barriers of delirium at the individual level."	Yes
1		Attitude 2.15, add the options "Nurses are busy, and delirium subtype assessment increases their workload," "Nurses do not have sufficient knowledge of delirium subtypes and assessment methods," and "Nurses do not know enough about delirium subtypes assessment tools and do not know how to assess them," "Nurses judge delirium subtypes based on clinical experience only, which is not accurate enough and cannot keep a written basis," "The department/hospital does not focus on delirium subtypes at present. No focus on delirium subtypes."	Yes
1		Practice 3.2.2 "What is the delirium assessment scale you use?" was replaced with the multiple-choice question "What is the most frequently used delirium assessment scale?"	Yes
1		Practice 3.3 add the option to "Ask a psychiatrist for assistance."	Yes
1	Add	Knowledge1.10 Adverse outcomes of delirium subtypes	Yes
1		Attitude 2.14: Nurses identify organizational-level barriers to delirium	Yes
1		Practice 3.5, which type of delirium is more common in clinical practice	Yes
1		knowledge sources 4.2.1, whether or not they passed the training test after attending the training	Yes
2	Modify	Knowledge 1.4, add the option "nursing factors: restraint, therapeutic manipulation, etc."	Yes
2		Attitude 2.16 option was amended to "No delirium subtype assessment at all."	Yes

**Table 6:** Clinical nurse delirium subtype assessment KAP status questionnaire.

Dimensions and Items			Importance		Rationality	
			Score ($\bar{X}$±s)	CV	Score ($\bar{X}$±s)	CV
Dimension 1:Knowledge						
	1.1	True or False: Delirium is an acute reversible mental disorder caused by various diseases.	4.90±0.32	0.06	4.90±0.32	0.06
	1.2	Multiple Choice: What are the risks of delirium? ⑴Increased mortality; ⑵Prolonged hospitalization; ⑶Increased hospitalization costs; ⑷Residual long-term perceptual impairment; ⑸Unawareness	5.00±0.00	0.00	5.00±0.00	0.00
	1.3	Multiple Choice: Which groups are at high risk for delirium? ⑴ICU patients; ⑵Post-operative patients; ⑶Elderly patients; ⑷Palliative care patients; ⑸Unawareness	5.00±0.00	0.00	5.00±0.00	0.00
	1.4	Multiple Choice: What are the risk factors for delirium? ⑴Patient factors: age, whether combined with underlying diseases, etc.; ⑵Drug factors: sedative drugs, analgesic drugs, etc.; ⑶Surgical factors: type of surgery, postoperative pain, etc.; ⑷Environmental factors: lights, machine alarms, etc.; ⑸Psychological factors: anxiety, depression, sense of stress, etc.; ⑹Nursing factors: restraint, therapeutic operations, etc.; ⑺Unawareness	5.00±0.00	0.00	4.90±0.32	0.06
	1.5	Multiple Choice: What are the clinical features of delirium? ⑴Inability to concentrate; ⑵Disorganized thinking; ⑶Increased activity; ⑷Decreased activity; ⑸Altered state of consciousness; ⑹Unawareness	5.00±0.00	0.00	5.00±0.00	0.00
	1.6	Multiple Choice: Which of the following tools are delirium assessment tools? ⑴DSM-5; ⑵ICD-10; ⑶CAM; ⑷CAM-ICU; ⑸ICDSC; ⑹Unawareness	4.90±0.32	0.06	4.90±0.32	0.06
	1.7	Multiple Choice: What are the management measures after the occurrence of delirium? ⑴Treatment of the cause; ⑵Intensive management; ⑶Early activity; ⑷Emphasis on sleep management; ⑸Targeted treatment according to different subtypes of delirium; ⑹Unawareness	5.00±0.00	0.00	4.90±0.32	0.06
	1.8	Multiple Choice: Key strategies to prevent and reduce delirium include which? ⑴Identify and modify risk factors that lead to delirium; ⑵Early detection of patients at risk for delirium; ⑶Pay attention to patients’ sleep management; ⑷Help patients at risk for delirium to perform early rehabilitation activities; ⑸Take timely restraint measures for patients with delirium; ⑹Unawareness	5.00±0.00	0.00	4.90±0.32	0.06
	1.9	Multiple choice: What is correct about the following clinical manifestations of each subtype of delirium? ⑴Hyporactive delirium is characterized by emotional poverty, indifference, drowsiness, and decreased reactivity; ⑵Hyperactive delirium is characterized by agitation, anxiety, and attempts to catch extubation; ⑶Mixed delirium shows fluctuations in agitation and quiet symptoms; ⑷Unawareness	4.90±0.32	0.06	4.80±0.42	.09
	1.10	Multiple Choice: What is correct about the following adverse outcomes for each subtype of delirium? ⑴Patients with increased activity delirium are relatively more likely to have adverse events such as falls, bed falls, and catheter extraction accidents; ⑵Patients with increased activity delirium are less likely to be detected by health care personnel; ⑶Patients with decreased activity delirium are more likely to have stress injuries; ⑷Decreased activity delirium is less likely to be noticed by health care personnel and has more severe effects on patients; ⑸It is not clear and cannot be judged	4.90±0.32	0.06	4.90±0.32	0.06
	1.11	Multiple Choice: Which of the following are delirium subtype assessment tools? ⑴ICDSC; ⑵RASS; ⑶DMSS; ⑷MDAS; ⑸Unawareness	4.40±0.70	0.16	4.50±0.71	0.16
Dimension 2:Attitude						
	2.1	Scale: How important do you think nursing care is in preventing and recovering delirium?	4.90±0.32	0.06	4.90±0.32	0.06
	2.2	Scale: Do you think clinical nurses should undertake the identification of delirium and delirium subtypes?	4.60±0.52	0.11	4.70±0.48	0.10
	2.3	Scale: Do you think clinical nurses should know delirium and delirium subtypes?	4.70±0.48	0.10	4.80±0.42	0.09
	2.4	Scale: Do you think your knowledge of delirium and delirium subtypes can meet clinical needs?	4.60±0.70	0.15	4.80±0.42	0.09
	2.5	Scale: Are you interested in knowledge about delirium and delirium subtypes?	4.70±0.48	0.10	4.50±0.53	0.12
	2.6	Scale: Do clinical nurses need to learn about delirium and its subtypes actively?	4.80±0.42	0.09	4.70±0.48	0.10
	2.7	Scale: Do you think clinical nurses must receive systematic training on knowledge related to delirium and delirium subtypes?	4.80±0.42	0.09	4.80±0.42	0.09
	2.8	Scale: Do you think conducting a delirium subtype assessment in clinical work is necessary?	4.80±0.42	0.09	4.80±0.42	0.09
	2.9	Scale: Do you think it is necessary to develop/introduce delirium subtype assessment tools?	5.00±0.00	0.00	5.00±0.00	0.00
	2.10	Scale: Are you willing to receive training on delirium subtypes?	4.70±0.48	0.10	4.80±0.42	0.09
	2.11	Multiple Choice: Which of the following types of delirium have you heard of? ⑴Hyperactive delirium; ⑵Hypoactive delirium ⑶Mixed delirium; ⑷Quiet delirium; ⑸Excited delirium; ⑹Depressed delirium; ⑺No motor delirium; ⑻Mixed delirium; ⑼Other ___________ (please fill in); ⑽ none of the above have been heard of	4.90±0.32	0.06	4.80±0.42	0.09
	2.12	Multiple Choice: What are your requirements for delirium assessment tools? ⑴Accurate assessment results; ⑵Rational assessment time; ⑶Rational frequency of assessment; ⑷Clear and easy to understand the text; ⑸Concise and clear forms; ⑹Other ___________ (please fill in)	5.00±0.00	0.00	5.00±0.00	0.00
	2.13	Multiple Choice: At the individual level, what are the current barriers to early delirium recognition for nurses? ⑴Insufficient knowledge base of delirium; ⑵Insufficient mastery of delirium assessment methods; ⑶Insufficient proficiency in the use of delirium assessment scales; ⑷Delirium assessment increases workload; ⑸Nurses are not confident enough in terms of their ability to assess delirium and do not trust the results of their assessment; ⑹Nurses are busy with clinical work and lack time to conduct delirium assessments; ⑺Nurses do not cooperate sufficiently with physicians; ⑻Other _________________(please fill in)	4.70±0.68	0.14	4.90±0.32	0.06
	2.14	Multiple Choice: At the organizational level, what are the current barriers to nurses’ early identification of delirium? ⑴The department/hospital does not have process specifications related to delirium assessment; ⑵The department/hospital does not conduct training related to delirium assessment; ⑶The department does not routinely conduct delirium assessments; ⑷The department does not provide delirium assessment tools; ⑸The department’s human resource allocation is inadequate; ⑹Other _________________(please fill in)	4.80±0.42	0.09	5.00±0.00	0.00
	2.15	Multiple Choice: In your opinion, what are the barriers to delirium subtype assessment? ⑴There is no significant difference in the clinical manifestations of each delirium subtype; ⑵There is no significant difference in the management measures of each delirium subtype; ⑶There is no significant difference in the prognostic impact of each delirium subtype; ⑷Nurses are busy with clinical work, and delirium subtype assessment will increase the workload of nurses; ⑸Nurses do not have enough knowledge of delirium subtypes and assessment methods; ⑹Nurses lack objective delirium subtype assessment tools; ⑺Delirium assessment work is still immature, and subtype assessment work is not carried out at all; ⑻The department/hospital does not focus on this part of the delirium subtype assessment at present and does not request the staff to assess the delirium subtype; ⑼Other __________(please fill in)	5.00±0.00	0.00	4.90±0.32	0.06
	2.16	Single Choice: How well do you think delirium assessment is done in the section you work in? (If it is convenient, please briefly describe the problems that exist) ⑴Very well done; ⑵Basically well done, but still some details are not enough __________; ⑶Not well done, still many problems ___________; ⑷No delirium assessment work at all	4.60±0.52	0.11	4.60±0.52	0.11
Dimension 3:Practice						
	3.1	Single Choice: In your daily clinical work, do you assess delirium? ⑴Always; ⑵Often; ⑶Sometimes; ⑷Sometimes; ⑸Never	5.00±0.00	0.00	4.90±0.32	0.06
	3.2	Single Choice: How do you assess and document delirium in your clinical work? ⑴Assessed by diagnostic scales and recorded; ⑵Assessed by diagnostic scales but not recorded; ⑶Assessed by clinical experience only and recorded; ⑷Assessed by clinical experience only and not recorded; ⑸ Did not assess delirium	5.00±0.00	0.00	5.00±0.00	0.00
		3.2.1 Single Choice: What do you record about delirium? (⑴ or ⑶ was selected for 3.2) A. "Patient has delirium of type xxx." B. "Patient has delirium." C. "Patient has confusion." D. "Patient has Behavioral mental abnormalities." E. Other, please describe _________________	4.90±0.32	0.06	4.80±0.42	0.09
		3.2.1.1 Multiple Choice: Why don’t you record this as "patient has delirium"? (C or D was selected for 3.2.1) (1) No delirium diagnostic tool was used for assessment; (2) Diagnostic tool was used but still not sure if the patient had delirium; (3) Physician did not make a diagnosis of delirium; (4) In the nursing records of the department, such patients were recorded as "confusion/abnormal mental behavior"; (5) Other _____ (please fill in)	4.90±0.32	0.06	4.90±0.32	0.06
		3.2.2 Single Choice: What is the scale you use most frequently? (⑴ or ⑵ was selected for 3.2) A.CAM; B.CAM-ICU; C.ICDSD; D.Nu-DESC; E.Other _____ (please fill in)	4.90±0.32	0.06	4.90±0.32	0.06
	3.3	Multiple Choice: How do you usually solve delirium problems when you encounter them in your daily clinical work? ⑴Discuss with doctors to solve the problem; ⑵Discuss with other nurses to solve the problem; ⑶Ask psychiatrists/psychologists to solve the problem; ⑷ Consult psychiatrists/psychologists; ⑸ Solve the problem independently; ⑹Other ___________(please fill in)	5.00±0.00	0.00	5.00±0.00	0.00
	3.4	Single Choice: In your daily clinical work, do you assess your patients’ type of delirium (delirium subtype)? ⑴always; ⑵often; ⑶sometimes; ⑷occasionally; ⑸ never	4.90±0.32	0.06	4.80±0.42	0.09
		3.4.1 Multiple Choice: Please tell me why you do not assess/are less likely to assess delirium subtypes? (⑶ or ⑷ or ⑸ was selected for 3.4) A. Do not know about delirium subtypes; B. Do not have delirium subtype assessment workers C. Do not know how to use delirium subtype assessment tools; D. Do not think the assessment is necessary; E. Other __________ (please fill in)	4.80±0.42	0.09	4.80±0.42	0.09
	3.5	Single Choice: In your daily clinical work, which of the following delirium patients are more common? ⑴Manic patients: increased speech, restlessness, and uncontrolled behavior; ⑵Quiet patients: slower movements, reduced speech, and lowered speaking volume; ⑶Mixed patients: alternation of the above two manifestations; ⑷I cannot distinguish between the above types of delirium patients	4.80±0.42	0.09	4.70±0.48	0.10
		3.5.1 Multiple Choice: How would you assess the patient’s delirium subtype? (⑴or ⑵ or ⑶ was selected for 3.5) A. By clinical experience; B. By assessing with the help of specific scales; C. By consulting with colleagues; D. Other ______ (please fill in)	4.80±0.42	0.09	4.80±0.42	0.09
		3.5.1.1 Fill in the blanks: I am using scale to assess delirium subtypes. (B was selected for 3.5.1)	4.60±0.70	0.15	4.50±0.71	0.16
Dimension 4:Knowledge sources						
	4.1	Scale: Does the knowledge you learned in school about delirium meet the needs of your current clinical work?	4.90±0.32	0.06	4.90±0.32	0.06
	4.2	Single Choice: Have you ever participated in delirium-related knowledge training? ⑴Yes; ⑵No	5.00±0.00	0.00	4.90±0.32	0.06
		4.2.1Single Choice: If you have participated in the training, at the end of the training, did you pass the training assessment? A. all passed; B. can pass about 80% or more of the assessment; C. can pass about 50%-80% of the assessment; D. can pass about 30%-50% of the assessment; E. can only pass about 30% of the assessment; F. no assessment session set	4.70±0.68	0.14	4.90±0.32	0.06
		4.2.2 Multiple Choice: Which of the following categories/does the knowledge training you attended belong to? A. Hospital level physician lectures (led by physicians/medical department, etc.); B. Hospital level nurse lectures (led by nurses or nursing department, etc.); C. Department level physician lectures (led by physician teaching team leader/director, etc.); D. Department level nurse lectures (led by nurse teaching team leader/nurse manager, etc.); E. Outbound training and learning; F. Participation in academic conferences; H. Personal initiative to learn relevant knowledge. I. Other ________(please fill in)	4.80±0.42	0.09	5.00±0.00	0.00
	4.3	Multiple Choice: Your knowledge of delirium and delirium subtypes comes primarily from the following sources?	4.90±0.32	0.06	4.90±0.32	0.06
	4.4	Multiple Choice: In what ways would you most like to enhance your knowledge about delirium and delirium subtypes?	5.00±0.00	0.06	5.00±0.00	0.00
		4.3&4.4 Options: ⑴ Study at school; ⑵ Academic conferences and lectures; ⑶ Relevant study classes; ⑷ Self-study (due to personal interest or work needs); ⑸ Work experience accumulation; ⑹ Exchange among colleagues; ⑺ Relevant media reports; ⑻ Consult relevant experts; ⑼ Brochures and publicity wall posters; ⑽ Others _______________(please fill in)				
	4.5	Multiple Choice: What are you most looking forward to learning about delirium and delirium subtypes? (Select up to 5 items) ⑴ Definition of delirium; ⑵ Monitoring and diagnosis of delirium; ⑶ Risk factors and etiology of delirium; ⑷ Prevention and management of delirium; ⑸ Definition and clinical manifestations of delirium subtypes; ⑹ Assessment methods and assessment tools of delirium subtypes; ⑺ Nursing measures and nursing priorities of delirium subtypes; ⑻ Other ______________(please fill in)	4.90±0.32	0.06	4.60±0.52	0.11

### 3.5 Validity and reliability test of the questionnaire

We tested the coefficient of internal consistency of the questionnaire through Cronbach’s alpha, which was 0.860, including all Likert-5-level scale items, including attitude, practice and source dimension. However, we didn’t involve in knowledge dimension because of questionnaire construction and question form. The Content Validity has been measured in the second round Delphi Consensus before, and all scores were acceptable, as shown in Table 6.

## 4. Discussion

Delirium is a transient state of consciousness characterized by confusion, commonly observed in clinical settings with a prevalence of up to one-third among hospitalized elderly aged 70 [13].It has been reported that mechanically ventilated patients in the ICU exhibit delirium at rates exceeding 75% in [23], while hip fracture repair and cardiac surgery patients experience delirium at rates surpassing 50% [24]. Therefore, delirium assessment has been noticed by researchers and medical staff around the world in these years. Previous studies have indicated that hyperactive delirious patients are more prone to falls within hospital or other environments, which may be associated with diminished quality of life [11, 25].

As the first caregiver for patients, nurses must promptly identify and accurately determine which delirium subtype exists at present in their patient. However, due to varying manifestations, each subtype receives different levels of attention, with hyperactive delirum being more easily detected and the mixed and hypoactive delirium being more easily ignored by health care professionals as previous researched have shown [12, 26]. This suggests that further clarification and quantification of nurses’ assessment ability regarding delirium subtypes.

Previous studies did not deepen this cognitive impairment into different subtypes perspectives, although DSM-5 has listed three delirium subtypes. A biliometric analysis revealed that there were only around 300 publications were available in past two decades [27]. Compared with previous studies regarding delirium knowledge assessment [28–30], we developed a novel measurement tool to evaluate nurses’ ability. The final nurses’ delirium subtypes assessment KAP questionnaire consisted of 37 items across four dimensions: delirium subtype-related knowledge, assessment attitude, assessment practice, and knowledge source. Our new questionnaire can give some revelations and deepen delirium research from the perspective of delirium subtypes. To our knowledge, this is the first questionnaire designed to quantitatively evaluate nurses’ ability regarding delirium subtypes assessment.

Our questionnaire has several advantages. Firstly, it enables nursing managers to visually assess nurses’ knowledge of each delirium subtype and quantitatively evaluate their clinical assessment behavior. Simultaneously, investigation of knowledge sources can provide valuable insights for nursing managers to supplement the delirium-related knowledge in a suitable and comprehensive way. In comparison with Yu’s questionnaire, ours not only measures the KAP level for all three delirium subtypes but also elucidates more details regarding assessment barriers details to enhance management of all three kinds of delirium subtypes, rather than solely hypoactive delirium [31].

Additionally, it has been observed that the majority of delirium research conducted among medical staff mainly focuses on a specific department, despite the fact that various patient populations may experience delirious [32, 33], such as those in intensive care units, elderly patients, post-operative patients, the dying patients and so on [34]. The lack of research addressing differences across departments has resulted in an unclear knowledge-attitude-practice (KAP) gap. Our questionnaire e aims to address this gap by providing a more comprehensive assessment tool applicable to clinical nurses working in diverse settings including general hospitals, psychiatric referral centers, community hospitals, and others. The construction process and item content of this questionnaire can also serve as a valuable reference for future investigations into the ability of clinicians and care workers to assess their ability regarding delirium subtype assessment across various medical locations.

Additionally, the development and validation of the questionnaire are grounded in scientific and theoretical principles. The initial draft of this questionnaire was formulated based on the KAP Model. Following discussion within the research team, we did not adopt all question as Liket-5-level scale format due to several considerations: 1) delirium subtypes are closely linked to the delirium section, it was not feasible to separate them; therefore, we a combination of multiple-choice and true-or-false questions for the knowledge section while reducing the number of knowledge-based questions by evaluating full-right, half-right, and incorrect responses; 2) in order to explore factors related to impairment in delirium assessment and subtypes assessment, we incorporated three multiple-choice questions in attitude dimension to decrease the question number; 3) aiming to gain insights into current practices and preferred tools among participants, logical-linked questions were included in the practice section. These modifications ensure that our questionnaire remains comprehensive while providing detailed investigation.

What’s more, Delphi method of expert correspondence was selected as the primary method for constructing the questionnaire in this study. Ten experts with rich theoretical knowledge and clinical experience in delirium and nursing management from various regions of China were chosen, who are familiar with clinical delirium assessment workflow, possess higher education, technical titles, and a broader age range to ensure adequate representation. Although Delphi studies typically involve 15 to 50 experts, only ten were included in this study due to the maturity of the KAP theory model used in the researches including the current status survey of a certain field. However, since this questionnaire was not conventional Likert-5-level format, it required input from experts on questions and options presented.

Although our questionnaire offers a novel perspective for assessing nurses’ delirium subtypes ability because it can use in different clinical situations based KAP model by Delphi method, there were still several limitations in this research. First, due to the special clinical enviorment in China, delirium is predominantly observed in general hospitals, we did not include experts from community hospital and elderly care facilities. Secondly, considering the number of questions involved and the complex content, we did not include reverse-scored questions in the questionnaire.

## 5. Conclusion

Guided by delirium-related guidelines for item content and the KAP theoretical model for the questionnaire constructure, we developed the assessment of clinical nurses’ delirium subtypes current status questionnaire based on the Delphi method. This scientifically and theoretically grounded development process ensured good reliability and content validity. The questionnaire incorporates reliable expert feedback and diverse question formats, enabling a multi-perspective evaluation of clinical nurses’ knowledge level and knowledge sources, assessment attitudes, assessment behaviors, and assessment barriers. Our questionnaire is comprehensive and detailed. In future study, the questionnaire will be distributed to clinical nurses in departments with a high incidence of delirium to validate further.

## Supporting information

S1 File (DOCX)

S2 File (DOCX)

S3 File (DOCX)

S4 File (DOCX)

S5 File (DOCX)
