# Supplementary material for: Development and validation of nurse’s assessment ability questionnaire in delirium subtypes: Based on Delphi expert consensus
Source: PLoS One. 2024 Jan 23;19(1):e0297063. doi: 10.1371/journal.pone.0297063 (PMC10805299; doi:10.1371/journal.pone.0297063)
Supplement: S1 File — (DOCX) [file pone.0297063.s001.docx]

**临床护士谵妄亚型评估的知信行现状调查 专家函询问卷（第一轮）**

尊敬的专家：

您好！首先衷心地感谢您愿意担任本项研究的函询专家！

我是重庆医科大学附属第二医院护理部甘秀妮教授的硕士研究生周雯，我们正在进行“临床护士谵妄亚型评估的知信行现状调查”研究。谵妄是一种急性临床综合征，可导致患者病死率增加、住院时间延长，引起长期的认知功能障碍，增加医疗费用，严重影响患者的预后。谵妄根据其临床特征不同可分为不同的亚型，各亚型有不同的临床表现和护理重点。本研究旨在调查临床护士谵妄及谵妄亚型评估的知识、态度、行为现状，以期了解目前临床护士的谵妄及谵妄亚型评估的现状，为后续选择合适的谵妄亚型评估工具为患者进行有效的谵妄亚型评估和针对性的护理措施提供参考。

在前期文献研究的基础上，现已形成了该问卷的初稿，包括一般资料部分、知识部分、态度部分、行为部分、知识来源部分5部分内容，在问卷内容上仍需您进一步指导。本次专家函询表包含两个部分：《专家基本信息调查表》和《“临床护士谵妄亚型评估的知信行现状调查”函询表》。对于您所提供的信息我们将严格保密，仅用于本次研究。

鉴于您在本领域具有较高的学术造诣和丰富的临床经验，恳切希望能够得到您的指导和帮助。您的意见将作为我们研究的重要依据！由于科研的时效性和研究进度的推进，恳请您于收到信件后**10个工作日内**回复意见和建议。如果您对问卷有任何疑问，请随时联系我们。

衷心地感谢您的支持和指导！祝您身体健康，工作顺利！

重庆医科大学附属第二医院

导师：甘秀妮 学生：周雯

联系人：周雯 联系方式：15123067794 邮箱：2020111843@stu.cqmu.edu.cn

**第一部分 专家基本信息表**

此表旨在了解您的基本情况，资料仅用于统计分析，绝对保密。请您根据个人实际情况填写，或在合适选项上打√或标红。如需进一步说明，请自行标注。

1. 姓名：
2. 性别：男 女
3. 年龄：
4. 最高学历：博士 硕士 本科 专科 其他
5. 工作单位：
6. 工作科室：
7. 工作年限：
8. 职称：正高级 副高级 中级 其他
9. 带教情况：博士生导师 硕士生导师 临床教学组长 临床带教老师 否
10. 主要研究方向：护理管理 护理教育 内科护理 外科护理 重症护理 其他
11. 联系方式： 电子邮箱：

**第二部分 “临床护士谵妄亚型评估的知信行现状调查”函询表**

**填表说明：**

1.本研究的调查对象是临床护士，即在各级医院从事临床护理工作的护士，不包括实习、请假的护士。

2.题目的重要性/合理性评分：非常重要/非常合理=5分，比较重要/比较合理=4分，一般=3分，不太重要/不太合理=2分，不重要/不合理=1分，请您在相应栏里打“√”或填写评分值。

3.如果您认为该题目描述不准确或应删除，请在“修改或删减意见”栏内填写修改内容或标注“删除”。

4.如果您认为我们还有未考虑到的需要增加的题目，请在“建议增加题目”空白栏内补充，并评价其重要程度与合理程度。

5.请勿缺项漏项。

**知识部分**

注：重要性评分/合理性评分：非常重要/非常合理=5分，比较重要/比较合理=4分，一般=3分，不太重要/不太合理=2分，不重要/不合理=1分

|  | **条目内容** | | **合理性评分** | | | | | **重要性评分** | | | | | | | | **修改或删除意见** |
| --- | --- | --- | --- | --- | --- | --- | --- | --- | --- | --- | --- | --- | --- | --- | --- | --- |
|  |  |  | **5** | **4** | **3** | **2** | **1** | **5** | **4** | | **3** | | **2** | | **1** |  |
| **谵妄**  **部分** | **1** | 请判断：谵妄是指由各种疾病引起的急性可逆性精神障碍。 [单选题] *A   \| A正确 B错误 C不了解 \| \| --- \| |  |  |  |  |  |  |  |  | |  | |  | |  |
|  | **2** | 下列哪类人群是谵妄发生的高危人群？ [多选题] *ABCD   \| A ICU患者 B 术后患者 C 老年患者 D 接受姑息治疗的患者 E 不了解 \| \| --- \| |  |  |  |  |  |  |  |  | |  | |  | |  |
|  | **3** | 谵妄可能的危险因素包括哪些？ [多选题] *ABCDE   \| A 患者因素 B 药物因素 C 手术因素 D 环境因素 E 医源性因素 F 不了解 \| \| --- \| |  |  |  |  |  |  |  |  | |  | |  | |  |
|  | **4** | 谵妄的临床特征包括哪些？ [多选题] *ABCDE   \| A 注意力不能集中 B 思维紊乱 C 活动增多 D 活动减少 E 意识状态改变 F 不了解 \| \| --- \| |  |  |  |  |  |  |  |  | |  | |  | |  |
|  | **5** | 下列哪些工具是谵妄评估/诊断工具？ [多选题] ABCD   \| A DSM-5 B ICD-10 C CAM D CAM-ICU E ICDSC F 不了解 \| \| --- \| |  |  |  |  |  |  |  |  | |  | |  | |  |
|  | **6** | 预防和减少谵妄的关键策略有哪些？ [多选题] *ABCDE   \| A 确定和改变导致谵妄的危险因素 B 及早发现谵妄高危患者  C 重视患者的睡眠管理 D 帮助谵妄高危患者进行早期康复活动 E 及时对高危患者进行亚型评估，有针对性地对不同类型的谵妄患者进行预防性处理 F 及时对谵妄患者采取约束措施 G 不了解 \| \| --- \| |  |  |  |  |  |  |  |  | |  | |  | |  |
|  | **7** | 谵妄的处理包括哪些？ [多选题] *ABCD   \| A 对因治疗 B 早期活动 C 重视睡眠管理 D 集束化管理 E 不了解 \| \| --- \| |  |  |  |  |  |  |  |  | |  | |  | |  |
|  | **8** | 谵妄的危害包括哪些？ [多选题] *ABCD   \| A 死亡率上升 B 住院时间延长 C 费用提高 D 遗留长期的知觉障碍 E 不了解 \| \| --- \| |  |  |  |  |  |  |  |  | |  | |  | |  |
| **谵妄亚型**  **部分** | **9** | 关于谵妄亚型，下列说法正确的是 [多选题] *ABCDE  A 活动减少型谵妄是以感情贫乏、感情淡漠、嗜睡和反应性降低为特征；  B 活动增多型谵妄是以躁动、焦虑并试图拔管为特征；  C 混合型谵妄表现为躁动与安静症状的波动；  D 活动增多谵妄更易发生不良事件，如跌倒、坠床、意外拔管；  E 活动减少型谵妄不易引起医护人员注意，对患者影响更为严重；  F 不了解 |  |  |  |  |  |  |  |  | |  | |  | |  |
|  | **10** | 您知道的谵妄亚型评估工具有哪些 [多选题] *BCD  A ICDSC B RASS C DMSS D MDAS E 其他 _________________ F 不清楚 |  |  |  |  |  |  |  |  | |  | |  | |  |
| **拟增加条目** |  |  |  |  |  |  |  |  |  |  | |  | |  | |  |
|  |  |  |  |  |  |  |  |  |  |  | |  | |  | |  |
|  |  |  |  |  |  |  |  |  |  |  | |  | |  | |  |
|  |  |  |  |  |  |  |  |  |  |  | |  | |  | |  |

**态度部分**

注：重要性评分/合理性评分：非常重要/非常合理=5分，比较重要/比较合理=4分，一般=3分，不太重要/不太合理=2分，不重要/不合理=1分

|  | **条目内容** | **合理性评分** | | | | | **重要性评分** | | | | | **修改或删除意见** |
| --- | --- | --- | --- | --- | --- | --- | --- | --- | --- | --- | --- | --- |
|  |  | **5** | **4** | **3** | **2** | **1** | **5** | **4** | **3** | **2** | **1** |  |
| **1** | 您认为护理工作对谵妄的预防及恢复有多重要？   \| ○非常重要 ○比较重要 ○一般 ○不是很重要 ○非常不重要 \| \| --- \| |  |  |  |  |  |  |  |  |  |  |  |
| **2** | 您认为护理人员是否应该承担谵妄及谵妄亚型的识别工作？   \| ○非常应该承担 ○比较应该承担 ○一般 ○不是很应该承担 ○非常不应该承担 \| \| --- \| |  |  |  |  |  |  |  |  |  |  |  |
| **3** | 您认为护理人员是否应该掌握谵妄及谵妄亚型的相关知识？   \| ○非常应该掌握 ○比较应该掌握 ○一般 ○不是很应该掌握 ○非常不应该掌握 \| \| --- \| |  |  |  |  |  |  |  |  |  |  |  |
| **4** | 您认为自身谵妄及谵妄亚型知识是否能够满足临床需要？  ○完全能够满足 ○比较能满足 ○一般 ○不是很满足 ○非常不满足 |  |  |  |  |  |  |  |  |  |  |  |
| **5** | 您是否对谵妄及谵妄亚型的相关知识感兴趣？  ○非常感兴趣 ○比较感兴趣 ○一般 ○不是很感兴趣 ○非常不感兴趣 |  |  |  |  |  |  |  |  |  |  |  |
| **6** | 您认为临床护士是否有必要主动学习谵妄及谵妄亚型相关知识？  ○非常有必要 ○有必要 ○一般，视个人情况 ○不是很必要 ○完全没必要 |  |  |  |  |  |  |  |  |  |  |  |
| **7** | 您认为有必要接受谵妄及谵妄亚型相关知识系统培训吗？  ○非常有必要 ○有必要 ○一般 ○不是很必要 ○完全没必要 |  |  |  |  |  |  |  |  |  |  |  |
| **8** | 以下类型的谵妄您曾听说过哪些？ [多选题] *   \| A 活动增多型谵妄 B 活动减少型谵妄 C 狂躁型谵妄 D 安静型谵妄 E 兴奋型谵妄  F 抑郁型谵妄 G 无活动型谵妄 H 混合型谵妄 I 其他 J 以上都没有听说过 \| \| --- \| |  |  |  |  |  |  |  |  |  |  |  |
| **9** | 您认为在您工作的科室中谵妄评估工作做得怎么样？（如果方便，请您简要描述存在的问题）  ○做得非常完善  ○做得基本完善，仍有些细节不足 （请描述：_________________ [选填]）  ○做得一般，仍有一些进步空间 （请描述：_________________ [选填]）  ○做得不太好，仍有很多问题 （请描述：_________________ [选填]）  ○完全没有评估谵妄 |  |  |  |  |  |  |  |  |  |  |  |
| **10** | 您认为目前影响护士早期识别谵妄的因素有哪些？ [多选题]  □谵妄知识不足  □人力资源不足  □与患者沟通较少  □谵妄多表现为活动减少型不易被发现  □缺乏适宜的评估工具  □缺乏系统的谵妄观察评估的流程、常规筛查、高危因素评估  □医护间合作不足  □其他 _________________ |  |  |  |  |  |  |  |  |  |  |  |
| **11** | 您认为有必要进行谵妄亚型评估吗？  ○非常有必要 ○很必要 ○一般 ○不是很必要 ○完全没必要 |  |  |  |  |  |  |  |  |  |  |  |
|  | **（选择“一般”、“不是很必要”、“完全没必要”）**  您认为“谵妄亚型评估必要性不大”的原因有哪些？[多选题]  □谵妄评估体系尚且不是非常成熟，亚型评估完全没有开展  □各谵妄亚型的临床表现没有太大差别  □各谵妄亚型的处理措施没有太大差别  □各谵妄亚型的对预后的影响没有太大差别  □人力资源配置不足  □缺乏适宜的评估工具  □护患沟通不足  □医护间合作不足  □其他 _________________ |  |  |  |  |  |  |  |  |  |  |  |
| **12** | 您认为有必要开发/引进谵妄亚型评估工具吗？  ○非常有必要 ○有必要 ○一般 ○不是很必要 ○完全没必要 |  |  |  |  |  |  |  |  |  |  |  |
| **13** | 您对谵妄亚型评估工具的要求有哪些？[多选题]  □文字清晰易懂  □评估结果准确  □评估用时合理  □评估频次合理  □表格简洁明了  □其他（请描述：_________________ [必填]） |  |  |  |  |  |  |  |  |  |  |  |
| **14** | 您愿意接受谵妄及谵妄亚型的相关知识培训吗？  ○非常愿意 ○比较愿意 ○一般 ○不太愿意 ○完全不愿意 |  |  |  |  |  |  |  |  |  |  |  |
| **拟增加条目** |  |  |  |  |  |  |  |  |  |  |  |  |

**行为部分**

注：重要性评分/合理性评分：非常重要/非常合理=5分，比较重要/比较合理=4分，一般=3分，不太重要/不太合理=2分，不重要/不合理=1分

|  | **条目内容** | **合理性评分** | | | | | **重要性评分** | | | | | **修改或删除意见** |
| --- | --- | --- | --- | --- | --- | --- | --- | --- | --- | --- | --- | --- |
|  |  | **5** | **4** | **3** | **2** | **1** | **5** | **4** | **3** | **2** | **1** |  |
| **1** | 请问您在临床工作中是否评估了谵妄？  ○总是 ○经常 ○有时 ○偶尔 ○从不 |  |  |  |  |  |  |  |  |  |  |  |
| **2** | 请问您在临床工作中是如何评估谵妄？ [单选题]  ○通过诊断量表进行评估，并记录  ○通过诊断量表进行评估，但未记录  ○仅靠临床经验进行评估，并记录  ○仅靠临床经验进行评估，并未记录  ○并未对谵妄进行评估 |  |  |  |  |  |  |  |  |  |  |  |
| **2.1** | **（选择“通过诊断量表进行评估”）**  请问您使用的量表是什么？[填空题]  **（选择“并记录”）**  请问关于谵妄您记录的内容是什么？[单选题]  ○“患者存在xxx型谵妄”  ○“患者存在谵妄”  ○“患者存在意识模糊”  ○“患者存在精神行为异常”  ○其他，请您描述 _________________ |  |  |  |  |  |  |  |  |  |  |  |
| **2.2** | **（选择“患者存在意识模糊”“患者存在精神行为异常”）**  请问您为什么不记录为“患者存在谵妄”？ [多选题]  □未使用谵妄诊断工具进行评估  □使用了诊断工具但仍不确定患者是否存在谵妄  □医生没有下谵妄诊断  □护理记录时科室默认此类患者均记录“意识模糊/精神行为异常”  □其他 _________________ |  |  |  |  |  |  |  |  |  |  |  |
| **3** | 请问您在临床工作中如果遇到谵妄问题一般怎么进行解决 [多选题]  □和医生商量解决  □和其他护士商量解决  □咨询精神科医生/心理科医生  □独立解决  □其他 _________________ |  |  |  |  |  |  |  |  |  |  |  |
| **4** | 请问您在临床工作中是否评估了患者谵妄的类型（谵妄亚型）？  ○总是 ○经常 ○有时 ○偶尔 ○从不 |  |  |  |  |  |  |  |  |  |  |  |
| **4.1** | **（选择“总是”“经常”“有时”“偶尔”）**  请问您如何评估患者谵妄的类型（谵妄亚型）？ [多选题] *  □通过临床经验评估  □通过借助某些量表评估  □通过与同事商量评估  □其他  **（选择“通过借助某些量表评估”）**  请问您借助的是什么量表来评估谵妄的类型（谵妄亚型）？ [填空题] _________________________________ |  |  |  |  |  |  |  |  |  |  |  |
| **4.2** | **（选择“有时”“偶尔”“从不”）**  请问您为何不评估/较少评估谵妄的类型（谵妄亚型）？ [多选题] *  □不了解谵妄亚型相关知识  □没有谵妄亚型评估工具  □不会使用谵妄亚型评估工具  □认为评估必要性不大  □其他 |  |  |  |  |  |  |  |  |  |  |  |
| **拟增加条目** |  |  |  |  |  |  |  |  |  |  |  |  |
|  |  |  |  |  |  |  |  |  |  |  |  |  |
|  |  |  |  |  |  |  |  |  |  |  |  |  |

**知识来源部分**

注：重要性评分/合理性评分：非常重要/非常合理=5分，比较重要/比较合理=4分，一般=3分，不太重要/不太合理=2分，不重要/不合理=1分

|  | **条目内容** | **合理性评分** | | | | | | **重要性评分** | | | | | **修改或删除意见** |
| --- | --- | --- | --- | --- | --- | --- | --- | --- | --- | --- | --- | --- | --- |
|  |  | **5** | **4** | **3** | **2** | **1** | **5** | | **4** | **3** | **2** | **1** |  |
| **1** | 您在学校所学的谵妄相关知识能否满足当前临床工作的需要？  ○完全能满足 ○比较能满足 ○一般 ○不太能满足 ○不能满足 |  |  |  |  |  |  | |  |  |  |  |  |
| **2** | 您是否参加过谵妄相关知识培训？  ○参加过10次以上（含10次） ○参加过5-9次 ○参加过2-4次 ○参加过1次 ○从未参加过 |  |  |  |  |  |  | |  |  |  |  |  |
| **3** | 您参加的知识培训属于以下哪个/哪些类别？ [多选题]  □医院层面医生讲座（由医生/医务科等牵头开展）  □医院层面护士讲座（由护士/护理部等牵头开展）  □科室层面医生讲座（由医生教学组长/主任等牵头开展）  □科室层面护士讲座（由护士教学组长/护士长等牵头开展）  □外出培训学习  □参加学术会议  □个人主动学习相关知识  □其他 |  |  |  |  |  |  | |  |  |  |  |  |
| **4** | 您的谵妄及谵妄亚型的知识主要来源于下列哪些途径？ [多选题]  □在校学习  □学术会议、讲座  □相关学习班  □自学（因个人兴趣或工作需要）  □工作经验积累  □同事之间交流  □相关媒体报道  □请教相关专家  □谵妄知识宣传册及宣传墙报  □其他 _________________ |  |  |  |  |  |  | |  |  |  |  |  |
| **5** | 你最希望通过哪些途径加强自己的谵妄及谵妄亚型相关知识 [多选题]  □在校学习  □学术会议、讲座  □相关学习班  □自学（因个人兴趣或工作需要）  □工作经验积累  □同事之间交流  □相关媒体报道  □请教相关专家  □宣传册及宣传墙报  □其他 _________________ |  |  |  |  |  |  | |  |  |  |  |  |
| **6** | 您当前最期望学习的谵妄及谵妄亚型的相关的知识有哪些？ [最多选5个]  □谵妄的定义  □谵妄的监测和诊断  □谵妄的危险因素和病因  □谵妄的病理生理  □谵妄各亚型的临床表现  □谵妄各亚型的评估方式  □谵妄各亚型的处理措施  □其他_________________ |  |  |  |  |  |  | |  |  |  |  |  |
| **拟增加条目** |  |  |  |  |  |  |  | |  |  |  |  |  |
|  |  |  |  |  |  |  |  | |  |  |  |  |  |
|  |  |  |  |  |  |  |  | |  |  |  |  |  |

请您选择本次调查中您的**判断依据及其影响程度**以及您对内容**的熟悉程度**

并在相应栏中打“√”

| **判断依据** | **影响程度** | | | | |
| --- | --- | --- | --- | --- | --- |
|  | **大** | **中** | | **小** | |
| **理论分析** |  |  | |  | |
| **实践经验** |  |  | |  | |
| **参考文献** |  |  | |  | |
| **直觉方面** |  |  | |  | |
| **您对本次调查内容的熟悉程度** | | | | | |
| **熟悉程度** | 很熟悉 | 比较熟悉 | 一般熟悉 | 不太熟悉 | 不熟悉 |
|  |  |  |  |  |  |
|  |  |  |  |  |  |

**问卷填写结束，衷心感谢您对本课题的支持与帮助！**

**祝您身体健康，生活愉快，工作顺利！**
