# Supplementary material for: Development and validation of nurse’s assessment ability questionnaire in delirium subtypes: Based on Delphi expert consensus
Source: PLoS One. 2024 Jan 23;19(1):e0297063. doi: 10.1371/journal.pone.0297063 (PMC10805299; doi:10.1371/journal.pone.0297063)
Supplement: S2 File — (DOCX) [file pone.0297063.s002.docx]

**临床护士谵妄亚型评估的知信行现状调查 专家函询问卷（第二轮）**

尊敬的专家：

您好！再次衷心地感谢您愿意担任本项研究的函询专家！

我是重庆医科大学附属第二医院护理部甘秀妮教授的硕士研究生周雯，我们正在进行“临床护士谵妄亚型评估的知信行现状调查”研究。非常感谢您在第一轮专家咨询中所提出的宝贵意见！

通过对第一轮结果的分析，综合各位专家的意见和建议，我们对“临床护士谵妄亚型评估的知信行现状调查”问卷进行了进一步的整理和归纳，我们尽量采纳了您在第一轮咨询中提出的宝贵意见，并对相应条目进行了调整。现将整理后的问卷内容反馈给您，请您再次进行评分并给予宝贵意见。本轮函询旨在对专家意见进行协调，请您对修改后的指标再次提出宝贵意见，这将对本研究中正式问卷的形成具有重要意义。

再次对您的指导表示感谢，恳请您于收到信件后**10个工作日内**回复意见和建议。

如果您对问卷有任何疑问，请随时联系我们。

衷心地感谢您的支持和指导！祝您身体健康，工作顺利！

重庆医科大学附属第二医院

导师：甘秀妮 学生：周雯

联系人：周雯 联系方式：15123067794 邮箱：2020111843@stu.cqmu.edu.cn

**第一部分 专家基本信息表**

此表旨在了解您的基本情况，资料仅用于统计分析，绝对保密。请您根据个人实际情况填写，或在合适选项上打√或标红。如需进一步说明，请自行标注。**若您第一轮已填写该部分，本轮可不填。**

1. 姓名：
2. 性别：男 女
3. 年龄：
4. 最高学历：博士 硕士 本科 专科 其他
5. 工作单位：
6. 工作科室：
7. 工作年限：
8. 职称：正高级 副高级 中级 其他
9. 带教情况：博士生导师 硕士生导师 临床教学组长 临床带教老师 否
10. 主要研究方向：护理管理 护理教育 内科护理 外科护理 重症护理 其他
11. 联系方式： 电子邮箱：

**第二部分 “临床护士谵妄亚型评估的知信行现状调查”函询表**

**填表说明：**

1.本研究的调查对象是临床护士，即在各级医院从事临床护理工作的护士，不包括实习、请假的护士。

2.新增或修改内容用**绿色底**标注，删除内容**已打删除线**

2.题目的重要性/合理性评分：非常重要/非常合理=5分，比较重要/比较合理=4分，一般=3分，不太重要/不太合理=2分，不重要/不合理=1分，请您在相应栏里打“√”或填写评分值。

3.如果您认为该题目描述不准确或应删除，请在“修改或删减意见”栏内填写修改内容或标注“删除”。

4.如果您认为我们还有未考虑到的需要增加的题目，请在“建议增加题目”空白栏内补充，并评价其重要程度与合理程度。

5.请勿缺项漏项。

**知识部分**

注：重要性评分/合理性评分：非常重要/非常合理=5分，比较重要/比较合理=4分，一般=3分，不太重要/不太合理=2分，不重要/不合理=1分

|  | **条目内容** | | **合理性评分** | | | | | **重要性评分** | | | | | | | | **修改或删除意见** |
| --- | --- | --- | --- | --- | --- | --- | --- | --- | --- | --- | --- | --- | --- | --- | --- | --- |
|  |  |  | **5** | **4** | **3** | **2** | **1** | **5** | **4** | | **3** | | **2** | | **1** |  |
| **谵妄**  **部分** | **1** | 请判断：谵妄是指由各种疾病引起的急性可逆性精神障碍。 [单选题] *A   \| A正确 B错误 C不了解 \| \| --- \| |  |  |  |  |  |  |  |  | |  | |  | |  |
|  | **2** | 谵妄的危害包括哪些？ [多选题] *ABCD   \| A 死亡率上升 B 住院时间延长 C 费用提高 D 遗留长期的知觉障碍 E 不了解 \| \| --- \| |  |  |  |  |  |  |  |  | |  | |  | |  |
|  | **3** | 下列哪类人群是谵妄发生的高危人群？ [多选题] *ABCD   \| A ICU患者 B 术后患者 C 老年患者 D 接受姑息治疗的患者 E 不了解 \| \| --- \| |  |  |  |  |  |  |  |  | |  | |  | |  |
|  | **4** | 谵妄可能的危险因素包括哪些？ [多选题] *ABCDE   \| A 患者因素：**年龄、是否合并基础疾病等** B 药物因素：**镇静药物、镇痛药物等**  C 手术因素：**手术类型、术后疼痛等** D 环境因素：**灯光、机器报警声等**  E心理因素：**焦虑、抑郁、压力感等** F 不了解  **选项增加了具体因素的举例** \| \| --- \| |  |  |  |  |  |  |  |  | |  | |  | |  |
|  | **5** | 谵妄的临床特征包括哪些？ [多选题] *ABCDE   \| A 注意力不能集中 B 思维紊乱 C 活动增多 D 意识状态改变 E 意识状态改变 F 不了解 \| \| --- \| |  |  |  |  |  |  |  |  | |  | |  | |  |
|  | **6** | **下列哪些工具是谵妄评估工具？（**原为：下列哪些工具为谵妄评估/诊断工具**）**[多选题] ABCDE   \| A DSM-5 B ICD-10 C CAM D CAM-ICU E ICDSC F 不了解 \| \| --- \| |  |  |  |  |  |  |  |  | |  | |  | |  |
|  | **7** | 谵妄的处理措施包括哪些？ [多选题] *ABCDE   \| A 对因治疗 B 早期活动 C 重视睡眠管理 D 集束化管理 **E 根据谵妄的不同亚型，进行针对性处理（新增选项）** F 不了解 \| \| --- \| |  |  |  |  |  |  |  |  | |  | |  | |  |
|  | **8** | 预防和减少谵妄的关键策略有哪些？ [多选题] *ABCD   \| A 确定和改变导致谵妄的危险因素 B 及早发现谵妄高危患者  C 重视患者的睡眠管理 D 帮助谵妄高危患者进行早期康复活动  E 及时对谵妄患者采取约束措施 F 不了解  **~~E 及时对高危患者进行亚型评估，有针对性地对不同类型的谵妄患者进行预防性处理~~ （删除该选项）** \| \| --- \| |  |  |  |  |  |  |  |  | |  | |  | |  |
| **谵妄亚型**  **部分** | **9** | **拆成定义和不良结局两个题**  **~~关于谵妄亚型，下列说法正确的是 [多选题] *ABCDE~~**  **~~A 活动减少型谵妄是以感情贫乏、感情淡漠、嗜睡和反应性降低为特征；~~**  **~~B 活动增多型谵妄是以躁动、焦虑并试图拔管为特征；~~**  **~~C 混合型谵妄表现为躁动与安静症状的波动；~~**  **~~D 活动增多谵妄更易发生不良事件，如跌倒、坠床、意外拔管；~~**  **~~E 活动减少型谵妄不易引起医护人员注意，对患者影响更为严重；~~**  **~~F 不了解~~**  **关于谵妄亚型的定义，下列说法正确的是 [多选题] *ABC**  A 活动减少型谵妄是以感情贫乏、感情淡漠、嗜睡和反应性降低为特征；  B 活动增多型谵妄是以躁动、焦虑并试图拔管为特征；  C 混合型谵妄表现为躁动与安静症状的波动；  **~~D 活动增多谵妄更易发生不良事件，如跌倒、坠床、意外拔管；~~**  **~~E 活动减少型谵妄不易引起医护人员注意，对患者影响更为严重；~~**  D 不了解 |  |  |  |  |  |  |  |  | |  | |  | |  |
|  | **10** | **关于谵妄亚型的不良结局，下列说法正确的是** [多选题] *ACD  **A活动增多型谵妄患者相对来说更易发生跌倒、坠床、导管拔出意外等不良事件；**  **B活动增多型谵妄患者更不容易被医护人员察觉；**  **C活动减少型谵妄患者更易发生压力性损伤；**  **D活动减少型谵妄不易引起医护人员注意，对患者影响更为严重；**  **E不清楚，无法判断** |  |  |  |  |  |  |  |  | |  | |  | |  |
|  | **11** | 您知道的谵妄亚型评估工具有哪些 [多选题] *BCD  A ICDSC B RASS C DMSS D MDAS E 其他 _________________ F 不清楚 |  |  |  |  |  |  |  |  | |  | |  | |  |
| **拟增加条目** |  |  |  |  |  |  |  |  |  |  | |  | |  | |  |
|  |  |  |  |  |  |  |  |  |  |  | |  | |  | |  |
|  |  |  |  |  |  |  |  |  |  |  | |  | |  | |  |
|  |  |  |  |  |  |  |  |  |  |  | |  | |  | |  |

**态度部分**

注：重要性评分/合理性评分：非常重要/非常合理=5分，比较重要/比较合理=4分，一般=3分，不太重要/不太合理=2分，不重要/不合理=1分

|  | **条目内容** | **合理性评分** | | | | | **重要性评分** | | | | | **修改或删除意见** |
| --- | --- | --- | --- | --- | --- | --- | --- | --- | --- | --- | --- | --- |
|  |  | **5** | **4** | **3** | **2** | **1** | **5** | **4** | **3** | **2** | **1** |  |
| **1** | 您认为护理工作对谵妄的预防及恢复有多重要？   \| ○非常重要 ○比较重要 ○一般 ○不是很重要 ○非常不重要 \| \| --- \| |  |  |  |  |  |  |  |  |  |  |  |
| **2** | 您认为临床护士是否应该承担谵妄及谵妄亚型的识别工作？   \| ○非常应该承担 ○比较应该承担 ○一般 ○不是很应该承担 ○非常不应该承担 \| \| --- \| |  |  |  |  |  |  |  |  |  |  |  |
| **3** | 您认为临床护士是否应该掌握谵妄及谵妄亚型的相关知识？   \| ○非常应该掌握 ○比较应该掌握 ○一般 ○不是很应该掌握 ○非常不应该掌握 \| \| --- \| |  |  |  |  |  |  |  |  |  |  |  |
| **4** | 您认为自身谵妄及谵妄亚型知识是否能够满足临床需要？  ○完全能够满足 ○比较能满足 ○一般 ○不是很满足 ○非常不满足 |  |  |  |  |  |  |  |  |  |  |  |
| **5** | 您是否对谵妄及谵妄亚型的相关知识感兴趣？  ○非常感兴趣 ○比较感兴趣 ○一般 ○不是很感兴趣 ○非常不感兴趣 |  |  |  |  |  |  |  |  |  |  |  |
| **6** | 您认为临床护士是否有必要主动学习谵妄及谵妄亚型相关知识？  ○非常有必要 ○有必要 ○一般，视个人情况 ○不是很必要 ○完全没必要 |  |  |  |  |  |  |  |  |  |  |  |
| **7** | 您认为临床护士有必要接受谵妄及谵妄亚型相关知识系统培训吗？  ○非常有必要 ○有必要 ○一般 ○不是很必要 ○完全没必要 |  |  |  |  |  |  |  |  |  |  |  |
| **8** | 您认为有必要进行谵妄亚型评估吗？  ○非常有必要 ○很必要 ○一般 ○不是很必要 ○完全没必要 |  |  |  |  |  |  |  |  |  |  |  |
| **9** | 您认为有必要开发/引进谵妄亚型评估工具吗？  ○非常有必要 ○有必要 ○一般 ○不是很必要 ○完全没必要 |  |  |  |  |  |  |  |  |  |  |  |
| **10** | 您愿意接受谵妄及谵妄亚型的相关知识培训吗？  ○非常愿意 ○比较愿意 ○一般 ○不太愿意 ○完全不愿意 |  |  |  |  |  |  |  |  |  |  |  |
| **11** | 以下类型的谵妄您曾听说过哪些？ [多选题] *   \| A 活动增多型谵妄 B 活动减少型谵妄 C 狂躁型谵妄 D 安静型谵妄 E 兴奋型谵妄 F 抑郁型谵妄 G 无活动型谵妄 H 混合型谵妄 I 其他 J 以上都没有听说过 （调整到态度部分） \| \| --- \| |  |  |  |  |  |  |  |  |  |  |  |
| **12** | 您对谵妄亚型评估工具的要求有哪些？ [多选题]  □文字清晰易懂  □评估结果准确  □评估用时合理  □评估频次合理  □表格简洁明了  □其他（请描述：_________________ [必填]） |  |  |  |  |  |  |  |  |  |  |  |
| **13** | **（原为：您认为目前影响护士早期识别谵妄的因素有哪些？）**  **改为2个题，从个人层面和组织层面分别提问障碍因素**  **新增：您认为，在个人层面，目前影响护士早期识别谵妄的障碍因素有哪些？ [多选题]**  □谵妄知识储备不足  □谵妄评估方法掌握不足  □谵妄评估量表使用不够熟练  □谵妄评估会增加工作量  □护士对自己谵妄评估的能力方面不够自信，不相信自己评估的结果  □护士临床工作繁忙，缺乏时间进行谵妄评估  □护士与医生合作不够充分  □其他 _________________ |  |  |  |  |  |  |  |  |  |  |  |
| **14** | **（新增）您认为，在组织层面，目前影响护士早期识别谵妄的障碍因素有哪些？**  □科室/医院没有谵妄评估的相关流程规范  □科室/医院没有开展谵妄评估的相关培训  □科室没有常规进行谵妄评估  □科室没有提供谵妄评估工具  □科室人力资源配置不足  □其他 _________________ |  |  |  |  |  |  |  |  |  |  |  |
| **15** | **~~（上一题选择“一般”、“不是很必要”、“完全没必要”）~~**  **去掉限定条件，改为必答题；修改表述，调整选项顺序**  **（原为：您认为“谵妄亚型评估必要性不大”的原因有哪些？）**  **您认为，谵妄亚型评估的障碍因素有哪些？[多选题]**  □各谵妄亚型的临床表现没有太大差别  □各谵妄亚型的处理措施没有太大差别  □各谵妄亚型的对预后的影响没有太大差别  **□护士的临床工作繁忙，谵妄亚型评估会增加护士的工作量（新增选项）**  **□护士对谵妄亚型知识和评估方法掌握不足（新增选项）**  **□护士不够了解谵妄亚型的评估工具，不知道该怎样进行评估（新增选项）**  □谵妄评估体系尚且不是非常成熟，亚型评估完全没有开展  **□护士仅根据临床经验判断谵妄亚型，不够准确，无法保留文字依据（新增选项）**  **□科室/医院现目前没有关注谵妄亚型的评估这部分内容（新增选项）**  □其他 _________________  **~~□人力资源配置不足~~（删除）**  **~~□缺乏适宜的评估工具~~（删除）**  **~~□护患沟通不足~~（删除）**  **~~□医护间合作不足~~（删除）** |  |  |  |  |  |  |  |  |  |  |  |
| **16** | 您认为在您工作的科室中谵妄评估工作做得怎么样？（如果方便，请您简要描述存在的问题）  ○做得非常完善  ○做得基本完善，仍有些细节不足 （请描述：_________________ [选填]）  **~~○做得一般，仍有一些进步空间 （请描述：_________________ [选填]~~ （与上一选项重复，删除）**  ○做得不太好，仍有很多问题 （请描述：_________________ [选填]）  **○完全没有开展谵妄亚型评估工作** |  |  |  |  |  |  |  |  |  |  |  |
| **拟增加条目** |  |  |  |  |  |  |  |  |  |  |  |  |
|  |  |  |  |  |  |  |  |  |  |  |  |  |

**行为部分**

注：重要性评分/合理性评分：非常重要/非常合理=5分，比较重要/比较合理=4分，一般=3分，不太重要/不太合理=2分，不重要/不合理=1分

|  | **条目内容** | **合理性评分** | | | | | **重要性评分** | | | | | **修改或删除意见** |
| --- | --- | --- | --- | --- | --- | --- | --- | --- | --- | --- | --- | --- |
|  |  | **5** | **4** | **3** | **2** | **1** | **5** | **4** | **3** | **2** | **1** | **/** |
| **1** | 在临床工作中，您是否进行了谵妄评估工作？  ○总是 ○经常 ○有时 ○偶尔 ○从不 |  |  |  |  |  |  |  |  |  |  |  |
| **2** | 请问您在临床工作中是如何评估谵妄？ [单选题]  ○通过诊断量表进行评估，并记录  ○通过诊断量表进行评估，但未记录  ○仅靠临床经验进行评估，并记录  ○仅靠临床经验进行评估，并未记录  ○并未对谵妄进行评估 |  |  |  |  |  |  |  |  |  |  |  |
| **2.1** | **（选择“并记录”）**  请问关于谵妄您记录的内容是什么？[单选题]  “患者存在xxx型谵妄”  ○“患者存在谵妄”  ○“患者存在意识模糊”  ○“患者存在精神行为异常”  ○其他，请您描述 _________________ |  |  |  |  |  |  |  |  |  |  |  |
| **2.1.1** | **（选择“患者存在意识模糊”“患者存在精神行为异常”）**  请问您为什么不记录为“患者存在谵妄”？ [**多选题**] *  □未使用谵妄诊断工具进行评估  □使用了诊断工具但仍不确定患者是否存在谵妄  □医生没有下谵妄诊断  □护理记录时科室默认此类患者均记录“意识模糊/精神行为异常”  □其他 _________________ |  |  |  |  |  |  |  |  |  |  |  |
| **2.2** | ~~请问您使用的量表是什么？[填空题]~~  **改为选择题**  （选择“通过诊断量表进行评估”），  **改为：请问您使用频率最高的量表是什么？** [单选题] *  ○CAM（意识模糊评估量表） ○CAM-ICU（ICU意识模糊评估量表）  ○ICDSC（ICU谵妄筛查量表） ○Nu-DESC（护理谵妄筛查量表） ○其他 _________________ |  |  |  |  |  |  |  |  |  |  |  |
| **3** | 在日常的临床工作中，您遇到谵妄问题一般怎么进行解决 [**多选题**] *  □和医生商量解决  □和其他护士商量解决  **□请精神科/心理科医生解决（新增选项）**  □咨询精神科/心理科医生  □独立解决  □其他 _________________ |  |  |  |  |  |  |  |  |  |  |  |
| **4** | 在日常的临床工作中，您是否评估了患者谵妄的类型（谵妄亚型）？[单选题]  ○总是 ○经常 ○有时 ○偶尔 ○从不 |  |  |  |  |  |  |  |  |  |  |  |
| **4.1** | **（选择“有时”“偶尔”“从不”）**  请问您为何不评估/较少评估谵妄的类型（谵妄亚型）？ [多选题] *  □不了解谵妄亚型相关知识 □没有谵妄亚型评估工具 □不会使用谵妄亚型评估工具  □认为评估必要性不大 □其他 |  |  |  |  |  |  |  |  |  |  |  |
| **5** | **（新增题目）在您日常的临床工作中，请问下列哪类谵妄患者更为多见？** [单选题] *  ○狂躁型患者：言语增多、躁动不安、行为失控  ○安静型患者：动作减缓、言语减少、说话音量降低  ○混合型患者：以上两种表现交替存在  ○我无法区分以上几类谵妄患者 |  |  |  |  |  |  |  |  |  |  |  |
| **5.1** | ~~（选择“总是”“经常”“有时”“偶尔”）~~  **限定条件改为“选择上一题目的前三个选项”**  请问您如何评估患者谵妄的类型（谵妄亚型）？ [**多选题**] *  □通过临床经验评估 □通过借助某些量表评估 □通过与同事商量评估 □其他 |  |  |  |  |  |  |  |  |  |  |  |
| **5.1.1** | **（选择“通过借助某些量表评估”）**  请问您借助的是什么量表来评估谵妄的类型（谵妄亚型）？ [**填空题**] _________________________________ |  |  |  |  |  |  |  |  |  |  |  |
| **拟增加条目** |  |  |  |  |  |  |  |  |  |  |  |  |
|  |  |  |  |  |  |  |  |  |  |  |  |  |

**知识来源部分**

注：重要性评分/合理性评分：非常重要/非常合理=5分，比较重要/比较合理=4分，一般=3分，不太重要/不太合理=2分，不重要/不合理=1分

|  |  | **合理性评分** | | | | | **重要性评分** | | | | | **修改或删除意见** |
| --- | --- | --- | --- | --- | --- | --- | --- | --- | --- | --- | --- | --- |
|  |  | **5** | **4** | **3** | **2** | **1** | **5** | **4** | **3** | **2** | **1** | **/** |
| **1** | 您在学校所学的谵妄相关知识能否满足当前临床工作的需要？  ○完全能满足 ○比较能满足 ○一般 ○不太能满足 ○不能满足 |  |  |  |  |  |  |  |  |  |  |  |
| **2** | 您是否参加过谵妄相关知识培训？  ○参加过 ○从未参加过  **（选项修改，去掉培训次数，增加考核通过率题目）**  （**~~原为：○参加过10次以上（含10次） ○参加过5-9次 ○参加过2-4次 ○参加过1次 ○从未参加过~~**） |  |  |  |  |  |  |  |  |  |  |  |
| **2.1** | **（第2题选择“参加过”）**  **在谵妄相关的培训结束后，请问您是否通过了培训考核？** [**单选题**] **（新增题目）**  ○全部通过  ○能够通过约80%以上的考核  ○能够通过约50%-80%的考核  ○能够通过约30%-50%的考核  ○仅能通过约30%的考核  ○没有设置考核环节 |  |  |  |  |  |  |  |  |  |  |  |
| **2.2** | （第2题选择“参加过”）  您参加的知识培训属于以下哪个/哪些类别？ [**多选题**]  □医院层面医生讲座（由医生/医务科等牵头开展）  □医院层面护士讲座（由护士/护理部等牵头开展）  □科室层面医生讲座（由医生教学组长/主任等牵头开展）  □科室层面护士讲座（由护士教学组长/护士长等牵头开展）  □外出培训学习  □参加学术会议  □个人主动学习相关知识  □其他 |  |  |  |  |  |  |  |  |  |  |  |
| **3** | 您的谵妄及谵妄亚型的知识主要来源于下列哪些途径？ [**多选题**]  □在校学习  □学术会议、讲座  □相关学习班  □自学（因个人兴趣或工作需要）  □工作经验积累  □同事之间交流  □相关媒体报道  □请教相关专家  □谵妄知识宣传册及宣传墙报  □其他 _________________ |  |  |  |  |  |  |  |  |  |  |  |
| **4** | 你最希望通过哪些途径加强自己的谵妄及谵妄亚型相关知识 [**多选题**]  □在校学习  □学术会议、讲座  □相关学习班  □自学（因个人兴趣或工作需要）  □工作经验积累  □同事之间交流  □相关媒体报道  □请教相关专家  □宣传册及宣传墙报  □其他 _________________ |  |  |  |  |  |  |  |  |  |  |  |
| **5** | 您当前最期望学习的谵妄及谵妄亚型的相关的知识有哪些？ [多选题] **（选项调整）**  □谵妄的定义  □谵妄的监测和诊断  □谵妄的危险因素和病因  □**谵妄的预防及处理（新增选项）**  □**~~谵妄的病理生理~~**  □**各个谵妄亚型的定义及临床表现（表述调整）**  □**各个谵妄亚型的评估方式及评估工具（表述调整）**  □**各个谵妄亚型的护理措施及护理重点（表述调整）**  □其他_________________ |  |  |  |  |  |  |  |  |  |  |  |
| **拟增加条目** |  |  |  |  |  |  |  |  |  |  |  |  |

请您选择本次调查中您的**判断依据及其影响程度**以及您对内容**的熟悉程度**

并在相应栏中打“√”

| **判断依据** | **影响程度** | | | | |
| --- | --- | --- | --- | --- | --- |
|  | **大** | **中** | | **小** | |
| **理论分析** |  |  | |  | |
| **实践经验** |  |  | |  | |
| **参考文献** |  |  | |  | |
| **直觉方面** |  |  | |  | |
| **您对本次调查内容的熟悉程度** | | | | | |
| **熟悉程度** | 很熟悉 | 比较熟悉 | 一般熟悉 | 不太熟悉 | 不熟悉 |
|  |  |  |  |  |  |
|  |  |  |  |  |  |

**问卷填写结束，衷心感谢您对本课题的支持与帮助！**

**祝您身体健康，生活愉快，工作顺利！**
