# Supplementary material for: Development and validation of nurse’s assessment ability questionnaire in delirium subtypes: Based on Delphi expert consensus
Source: PLoS One. 2024 Jan 23;19(1):e0297063. doi: 10.1371/journal.pone.0297063 (PMC10805299; doi:10.1371/journal.pone.0297063)
Supplement: S3 File — (DOCX) [file pone.0297063.s003.docx]

# Delphi Consensus of *Development of the clinical nurse delirium subtype assessment KAP status questionnaire* (First Round)

Dear Professors,

Thanks sincerely for your willingness to serve as a correspondence expert for this study.

I am Wen Zhou, a nursing master student from the Second Hospital of Chongqing Medical University. My tutor is Xiuni Gan, the Chief of Nursing Department. We are conducting a study regarding the current knowledge, attitude and practice status of delirium subtypes assessment among clinical nurses.

Delirium is an acute clinical syndrome that can lead to increased mortality, prolonged hospitalization, long-term cognitive impairment, increased medical costs, and serious impact on patient prognosis. Delirium can be divided into different subtypes according to its clinical features, each subtype has different clinical manifestations and nursing priorities. The aim of this study is to clarify the current status of delirium and delirium subtype assessment among clinical nurses, which will provide helpful suggestions for the subsequent delirium subtypes management and targeted nursing measures for delirium patients.

Based on the preliminary literature study, the first draft of KAP questionnaire has now been formed, including five parts: general information section, knowledge section, attitude section, behavior section, and knowledge source section, which still needs your further guidance on the content of the questionnaire.

The expert questionnaire consists of two parts, Part 1: *Experts basic information questionnaire*; Part 2: *Consensus Questionnaire of Clinical nurse delirium subtype assessment Knowledge-Attitude-Practice status questionnaire*. The information provided by you will be kept strictly confidential and used only for this study.

We hope to receive your guidance and assistance sincerely given your high academic attainment and rich clinical experience in delirium field. Your opinion will serve as an important basis for our research. Due to the timeliness and the progress, we kindly ask you to reply to the comments within 10 weekdays after receiving the letter. If you have any questions about the questionnaire, please feel free to contact us.

Thank you for your support and guidance sincerely.

Best Wishes to you!

The Second Affiliated Hospital of Chongqing Medical University

Tutor: Xiuni Gan

Student: Wen Zhou

Contacts：Wen Zhou Phone / Wechat：15123067794 E-mail：[631269011@qq.com](mailto:631269011@qq.com)

## Part 1 Experts basic information questionnaire

This questionnaire is designed to understand your situation. The information is only used for statistical analysis, absolute confidentiality, and never for other purposes. Please fill in the form according to your actual situation, and mark the "√" behind the indicator or mark the appropriate option in red. If you need further explanation, please mark the corresponding column.

1. Name:

2. Gender: Man Woman

3. Age:

4. Highest degree: Doctor Master Bachelor College Others:

5. Work Organization

6. Work Department

7. Work Duration

8. Technical Title: Senior Senior Vice Moderate Others:

9. Tutor: Doctor Tutor Master Tutor No

10. Research Direction: Nursing management Nursing Education Medical Nursing Surgical Nursing Critical Nursing Others:

11. Contact: Phone: ; E-mail:

## Part 2: Consensus Questionnaire of *Clinical nurse delirium subtype assessment Knowledge-Attitude-Practice status questionnaire*

Questionnaire introduction:

1. The target participants are clinical nurses who are working in a clinically responsible nursing position in all types hospital, the nurses who are on the sick leave or maternity leave, or interns were excluded.

2. Rationality Score and Importance Score: 5 points = Very rational/ Very important; 4 points = More rational/ More important; 3 points = General rational/ General important; 2 points = Not very rational/ Not very important; 1 point = Strongly not rational; Strongly not important.

3. If you believe that the description of the content is inaccurate or should be deleted, please fill in the "Comments for amendment or deletion" field or indicate "Delete".

4. If you think there are additional content that we have not considered, please add them in the "Suggested additions" blank box, and please judge the importance of the changes and additions in the same way.

5. Please fill in all items.

### Knowledge Section

5 points = Very rational/ Very important; 4 points = More rational/ More important; 3 points = Generally rational/ Generally important; 2 points = Not very rational/ Not very important; 1 point = Strongly not rational; Strongly not important

|  | **Content** | | **Rationality Score** | | | | | **Importance Score** | | | | | | | | **Expert opinions** |
| --- | --- | --- | --- | --- | --- | --- | --- | --- | --- | --- | --- | --- | --- | --- | --- | --- |
|  |  |  | **5** | **4** | **3** | **2** | **1** | **5** | **4** | | **3** | | **2** | | **1** |  |
| Delirium  Part | **1** | True or False: Delirium is an acute reversible mental disorder caused by various diseases.*1   \| ⑴True ⑵False ⑶I don't know \| \| --- \| |  |  |  |  |  |  |  |  | |  | |  | |  |
|  | **2** | Multiple Choice: Which groups are at high risk for delirium?*1234  ⑴ICU patients; ⑵Post-operative patients; ⑶Elderly patients; ⑷Palliative care patients; ⑸I don't know |  |  |  |  |  |  |  |  | |  | |  | |  |
|  | 3 | Multiple Choice: What are the risk factors for delirium?*12345  ⑴Patient factors ⑵Drug factors ⑶Surgical factors ⑷Environmental factors ⑸Psychological factors ⑹I don't know |  |  |  |  |  |  |  |  | |  | |  | |  |
|  | **4** | \| Multiple Choice: What are the clinical features of delirium?*12345  ⑴Inability to concentrate;  ⑵Disorganized thinking;  ⑶Increased activity;  ⑷Decreased activity;  ⑸Altered state of consciousness;  ⑹I don't know \| \| --- \| |  |  |  |  |  |  |  |  | |  | |  | |  |
|  | **5** | Multiple Choice: Which of the following tools are delirium screening tools or delirium diagnosis tools?*****1234   \| ⑴DSM-5; ⑵ICD-10; ⑶CAM; ⑷CAM-ICU; ⑸ICDSC; ⑹I don't know \| \| --- \| |  |  |  |  |  |  |  |  | |  | |  | |  |
|  | **6** | Multiple Choice: Key strategies to prevent and reduce delirium include which?*12345  ⑴Identify and modify risk factors that lead to delirium;  ⑵Early detection of patients at risk for delirium;  ⑶Pay attention to patients' sleep management;  ⑷Timely subtype assessment of high-risk patients and targeted preventive treatment for different types of delirium  ⑸Help patients at risk for delirium to perform early rehabilitation activities  ⑹Take timely restraint measures for patients with delirium;  ⑺I don't know |  |  |  |  |  |  |  |  | |  | |  | |  |
|  | **7** | Multiple Choice: What are the management measures after the occurrence of delirium?*1234  ⑴Treatment of the cause;  ⑵Early activity;  ⑶Emphasis on sleep management;  ⑷Intensive management;  ⑸I don't know |  |  |  |  |  |  |  |  | |  | |  | |  |
|  | **8** | Multiple Choice: What are the risks of delirium?*1234  ⑴Increased mortality;  ⑵Prolonged hospitalization;  ⑶Increased hospitalization costs;  ⑷Residual long-term perceptual impairment;  ⑸I don't know |  |  |  |  |  |  |  |  | |  | |  | |  |
| Delirium Subtypes Part | **9** | Multiple Choice: What is correct about the following selection of delirium subtypes? *12345  ⑴Hypoactive delirium is characterized by emotional poverty, indifference, drowsiness, and decreased reactivity;  ⑵Hyperactive delirium is characterized by agitation, anxiety, and attempts to catch extubation;  ⑶Mixed delirium shows fluctuations in agitation and quiet symptoms;  ⑷Patients with increased activity delirium are relatively more likely to have adverse events such as falls, bed falls, and catheter extraction accidents;  ⑸Decreased activity delirium is less likely to be noticed by health care workers and has a more serious impact on patients;  ⑹I don’t know. |  |  |  |  |  |  |  |  | |  | |  | |  |
|  | **10** | Multiple Choice: Which of the following are delirium subtype assessment tools?*234  ⑴ICDSC; ⑵RASS; ⑶DMSS; ⑷MDAS; ⑸I don't know |  |  |  |  |  |  |  |  | |  | |  | |  |
| **Addition** |  |  |  |  |  |  |  |  |  |  | |  | |  | |  |
|  |  |  |  |  |  |  |  |  |  |  | |  | |  | |  |

### Attitude Section

5 points = Very rational/ Very important; 4 points = More rational/ More important; 3 points = Generally rational/ Generally important; 2 points = Not very rational/ Not very important; 1 point = Strongly not rational; Strongly not important

|  | **Content** | **Rationality Score** | | | | | **Importance Score** | | | | | **Expert opinions** |
| --- | --- | --- | --- | --- | --- | --- | --- | --- | --- | --- | --- | --- |
|  |  | **5** | **4** | **3** | **2** | **1** | **5** | **4** | **3** | **2** | **1** |  |
| **1** | Scoring: How important do you think nursing care is in preventing and recovering delirium? |  |  |  |  |  |  |  |  |  |  |  |
| **2** | Scoring: Do you think clinical nurses should undertake the identification of delirium and delirium subtypes? |  |  |  |  |  |  |  |  |  |  |  |
| **3** | Scoring: Do you think clinical nurses should know delirium and delirium subtypes? |  |  |  |  |  |  |  |  |  |  |  |
| **4** | Scoring: Do you think your knowledge of delirium and delirium subtypes can meet clinical needs? |  |  |  |  |  |  |  |  |  |  |  |
| **5** | Scoring: Are you interested in knowledge about delirium and delirium subtypes? |  |  |  |  |  |  |  |  |  |  |  |
| **6** | Scoring: Do clinical nurses need to learn about delirium and its subtypes actively? |  |  |  |  |  |  |  |  |  |  |  |
| **7** | Scoring: Do you think clinical nurses must receive systematic training on knowledge related to delirium and delirium subtypes? |  |  |  |  |  |  |  |  |  |  |  |
| **8** | Multiple Choice: Which of the following types of delirium have you heard of?  ⑴Hyperactive delirium;  ⑵Hypoactive delirium  ⑶Mixed delirium;  ⑷Quiet delirium;  ⑸Excited delirium;  ⑹Depressed delirium;  ⑺No motor delirium;  ⑻Mixed delirium;  ⑼Other ___________ (please fill in);  ⑽ none of the above have been heard of |  |  |  |  |  |  |  |  |  |  |  |
| **9** | Single Choice: How well do you think delirium assessment is done in the section you work in? (If it is convenient, please briefly describe the problems that exist)  ⑴Very well done;  ⑵Basically well done, but still some details are not enough (please describe it: __________optional blank);  ⑶Generally done, but still need to improve (please describe it: __________optional blank);  ⑷Not well done, still many problems (please describe it: __________optional blank);  ⑸No delirium assessment work at all |  |  |  |  |  |  |  |  |  |  |  |
| **10** | Multiple Choice: What are the current barriers to early delirium recognition for nurses?  ⑴Insufficient knowledge base of delirium;  ⑵The department's human resource allocation is inadequate;  ⑶Nurses have little communication with patients  ⑷Delirium is mostly manifested as decreased activity and is not easy to be noticed;  ⑸There is no suitable assessment tool in clinical situation;  ⑹The department/hospital does not have process specifications related to delirium assessment;  ⑺Nurses do not cooperate sufficiently with physicians;  ⑻Other barriers _________________ |  |  |  |  |  |  |  |  |  |  |  |
| **11** | Scoring: Do you think conducting a delirium subtype assessment in clinical work is necessary? |  |  |  |  |  |  |  |  |  |  |  |
|  | （If you score 1 to 3 points, please answer following question)  Why do you think it is not necessary to assess delirium subtypes?  ⑴Delirium assessment work is still immature, and subtype assessment work is not carried out at all;  ⑵There is no significant difference in the clinical manifestations of each delirium subtype;  ⑶There is no significant difference in the management measures of each delirium subtype;  ⑷There is no significant difference in the prognostic impact of each delirium subtype;  ⑸The department's human resource allocation is inadequate;  ⑹The department does not provide delirium assessment tools;  ⑺Nurses have little communication with patients  ⑻Nurses do not cooperate sufficiently with physicians;  ⑼Other barriers _________________ |  |  |  |  |  |  |  |  |  |  |  |
| **12** | Scoring: Do you think it is necessary to develop/introduce delirium subtype assessment tools? |  |  |  |  |  |  |  |  |  |  |  |
| **13** | Multiple Choice: What are your requirements for delirium assessment tools?  ⑴Accurate assessment results;  ⑵Reasonable assessment time;  ⑶Reasonable frequency of assessment;  ⑷Clear and easy to understand the text;  ⑸Concise and clear forms;  ⑹Other ___________ (please fill in) |  |  |  |  |  |  |  |  |  |  |  |
| **14** | Scoring: Are you willing to receive training on delirium subtypes? |  |  |  |  |  |  |  |  |  |  |  |
| **Addition** |  |  |  |  |  |  |  |  |  |  |  |  |
|  |  |  |  |  |  |  |  |  |  |  |  |  |

### Practice Section

5 points = Very rational/ Very important; 4 points = More rational/ More important; 3 points = Generally rational/ Generally important; 2 points = Not very rational/ Not very important; 1 point = Strongly not rational; Strongly not important

|  | **Content** | **Rationality Score** | | | | | **Importance Score** | | | | | **Expert opinions** |
| --- | --- | --- | --- | --- | --- | --- | --- | --- | --- | --- | --- | --- |
|  |  | **5** | **4** | **3** | **2** | **1** | **5** | **4** | **3** | **2** | **1** |  |
| **1** | Single Choice： In your daily clinical work, do you assess delirium?  ⑴Always; ⑵Often; ⑶Sometimes; ⑷Sometimes; ⑸Never |  |  |  |  |  |  |  |  |  |  |  |
| **2** | Single Choice： How do you assess and document delirium in your clinical work?  ⑴Assessed by diagnostic scales and recorded;  ⑵Assessed by diagnostic scales but not recorded;  ⑶Assessed by clinical experience only and recorded;  ⑷Assessed by clinical experience only and not recorded;  ⑸ Did not assess delirium |  |  |  |  |  |  |  |  |  |  |  |
| **2.1** | （If you selected (1) or (2), please answer following questions)  Which scales did you use? Please fill in the blank.  （If you selected (1) or (3), please answer following questions)  Single Choice: What do you record about delirium? (⑴ or ⑶ was selected for 3.2)  A. "Patient has delirium of type xxx."  B. "Patient has delirium."  C. "Patient has confusion."  D. "Patient has Behavioral mental abnormalities."  E. Other, please describe _________________ |  |  |  |  |  |  |  |  |  |  |  |
| **2.2** | Multiple Choice: Why don't you record this as "patient has delirium"? ( C or D was selected for 3.2.1)  (1) No delirium diagnostic tool was used for assessment;  (2) Diagnostic tool was used but still not sure if the patient had delirium;  (3) Physician did not make a diagnosis of delirium;  (4) In the nursing records of the department, such patients were recorded as "confusion/abnormal mental behavior";  (5) Other _____ (please fill in) |  |  |  |  |  |  |  |  |  |  |  |
| **3** | Multiple Choice: How do you usually solve delirium problems when you encounter them in your daily clinical work?  ⑴Discuss with doctors to solve the problem;  ⑵Discuss with other nurses to solve the problem;  ⑶Consult psychiatrists/psychologist;  ⑷ Solve the problem independently;  ⑸ Other ___________(please fill in) |  |  |  |  |  |  |  |  |  |  |  |
| **4** | In your daily clinical work, do you assess your patients' type of delirium (delirium subtype)?  ⑴always; ⑵often; ⑶sometimes; ⑷occasionally; ⑸ never |  |  |  |  |  |  |  |  |  |  |  |
| **4.1** | （If you selected (1) (2) (3)of 4^nd^ question, please answer following questions)  How would you assess the patient's delirium subtype?  A. By clinical experience;  B. By assessing with the help of specific scales;  C. By consulting with colleagues;  D. Other ______ (please fill in)  （If you selected B, please answer following questions)  Fill in the blanks: I am using scale to assess delirium subtypes. |  |  |  |  |  |  |  |  |  |  |  |
| **4.2** | （If you selected (3) (4) (5)of 4^nd^ question, please answer following questions)  Please tell me why you do not assess/are less likely to assess delirium subtypes?  A. Do not know about delirium subtypes;  B. Do not have delirium subtype assessment workers  C. Do not know how to use delirium subtype assessment tools;  D. Do not think the assessment is necessary;  E. Other __________ (please fill in) |  |  |  |  |  |  |  |  |  |  |  |
| **Addition** |  |  |  |  |  |  |  |  |  |  |  |  |
|  |  |  |  |  |  |  |  |  |  |  |  |  |

### Knowledge Source Section

5 points = Very rational/ Very important; 4 points = More rational/ More important; 3 points = Generally rational/ Generally important; 2 points = Not very rational/ Not very important; 1 point = Strongly not rational; Strongly not important

|  | **Content** | **Rationality Score** | | | | | **Importance Score** | | | | | **Expert opinions** |
| --- | --- | --- | --- | --- | --- | --- | --- | --- | --- | --- | --- | --- |
|  |  | **5** | **4** | **3** | **2** | **1** | **5** | **4** | **3** | **2** | **1** | **/** |
| **1** | Scoring: Does the knowledge you learned in school about delirium meet the needs of your current clinical work? |  |  |  |  |  |  |  |  |  |  |  |
| **2** | Single Choice: Have you ever participated in delirium-related knowledge training?  ⑴More than 10 times training( including 10); ⑵5 to 9 times training; ⑶2 to 4 times training; ⑷only once; ⑸no training |  |  |  |  |  |  |  |  |  |  |  |
| **3** | Multiple Choice: Which of the following categories/does the knowledge training you attended belong to?  A. Hospital level physician lectures (led by physicians/medical department, etc.);  B. Hospital level nurse lectures (led by nurses or nursing department, etc.);  C. Department level physician lectures (led by physician teaching team leader/director, etc.);  D. Department level nurse lectures (led by nurse teaching team leader/nurse manager, etc.);  E. Outbound training and learning;  F. Participation in academic conferences;  H. Personal initiative to learn relevant knowledge.  I. Other ________(please fill in) |  |  |  |  |  |  |  |  |  |  |  |
| **4** | Multiple Choice: Which of the following sources your knowledge of delirium and delirium subtypes comes primarily from ?  ⑴ Study at school;  ⑵ Academic conferences and lectures;  ⑶ Relevant study classes;  ⑷ Self-study (due to personal interest or work needs);  ⑸ Work experience accumulation;  ⑹ Exchange among colleagues;  ⑺ Relevant media reports;  ⑻ Consult relevant experts;  ⑼ Brochures and publicity wall posters;  ⑽ Others _______________(please fill in) |  |  |  |  |  |  |  |  |  |  |  |
| **5** | Multiple Choice: In what ways would you most like to enhance your knowledge about delirium and delirium subtypes?  ⑴ Study at school;  ⑵ Academic conferences and lectures;  ⑶ Relevant study classes;  ⑷ Self-study (due to personal interest or work needs);  ⑸ Work experience accumulation;  ⑹ Exchange among colleagues;  ⑺ Relevant media reports;  ⑻ Consult relevant experts;  ⑼ Brochures and publicity wall posters;  ⑽ Others _______________(please fill in) |  |  |  |  |  |  |  |  |  |  |  |
| **6** | Multiple Choice: What are you most looking forward to learning about delirium and delirium subtypes? (Select up to 5 items)  ⑴ Definition of delirium;  ⑵ Monitoring and diagnosis of delirium;  ⑶ Risk factors and etiology of delirium;  ⑷ Pathophysiology of delirium;  ⑸ Definition and clinical manifestations of delirium subtypes;  ⑹ Assessment methods and assessment tools of delirium subtypes;  ⑺ Nursing measures and nursing priorities of delirium subtypes;  ⑻ Other ______________(please fill in) |  |  |  |  |  |  |  |  |  |  |  |
| **Addition** |  |  |  |  |  |  |  |  |  |  |  |  |
|  |  |  |  |  |  |  |  |  |  |  |  |  |

Please select the basis for your judgement, level of influence and familiarity with the above entry “√” the appropriate column

| Basis of judgement | Level of influence | | | | |
| --- | --- | --- | --- | --- | --- |
|  | High | middle | | Low | |
| Theoretical analysis |  |  | |  | |
| Practical experience |  |  | |  | |
| Literature reading |  |  | |  | |
| Intuitive feeling |  |  | |  | |
| How familiar are you with the content of this survey | | | | | |
| Degree of familiarity | Extremely familiar | Very familiar | Generally familiar | Slightly familiar | Unfamiliar |
|  |  |  |  |  |  |

End of form. Thank you again for your support and help with this subject.

I wish you a happy life. Good luck with your work.
