# Supplementary material for: Development and validation of nurse’s assessment ability questionnaire in delirium subtypes: Based on Delphi expert consensus
Source: PLoS One. 2024 Jan 23;19(1):e0297063. doi: 10.1371/journal.pone.0297063 (PMC10805299; doi:10.1371/journal.pone.0297063)
Supplement: S4 File — (DOCX) [file pone.0297063.s004.docx]

# Delphi Consensus of *Development of the clinical nurse delirium subtype assessment KAP status questionnaire* (Second Round)

Dear Professors,

Thanks sincerely for your willingness to serve as a correspondence expert for this study again. We are so appreciated to your helpful and constructive suggestions in the first Delphi Consensus round.

I am Wen Zhou, a nursing master student from the Second Hospital of Chongqing Medical University. My tutor is Xiuni Gan, the Chief of Nursing Department. We are conducting a study regarding the current knowledge, attitude and practice status of delirium subtypes assessment among clinical nurses.

Through the analysis of the results of the first round, we have further organized and summarized the experts’ suggestions regarding the questionnaire *Clinical nurse delirium subtype assessment Knowledge-Attitude-Practice status questionnaire*. Now, we are going to give you back the contents of the analysis. If you could rate it again and give us your valuable opinions, we will so appreciate it. The purpose of the Second Round is to coordinate the expert opinions. Your suggestions are important for the formation of the formal questionnaire in this study. Due to the timeliness and the progress, we kindly ask you to reply to the comments within 10 weekdays after receiving the letter. If you have any questions about the questionnaire, please feel free to contact us.

Thank you again for your support and guidance sincerely.

Best Wishes to you!

The Second Affiliated Hospital of Chongqing Medical University

Tutor: Xiuni Gan

Student: Wen Zhou

Contacts：Wen Zhou Phone / Wechat：15123067794 E-mail：[631269011@qq.com](mailto:631269011@qq.com)

## Part 1 Experts basic information questionnaire

This questionnaire is designed to understand your situation. The information is only used for statistical analysis, absolute confidentiality, and never for other purposes. Please fill in the form according to your actual situation, and mark the "√" behind the indicator or mark the appropriate option in red. If you need further explanation, please mark the corresponding column. If you have filled it in the first round, it is acceptable to be blank.

1. Name:

2. Gender: Man Woman

3. Age:

4. Highest degree: Doctor Master Bachelor College Others:

5. Work Organization

6. Work Department

7. Work Duration

8. Technical Title: Senior Senior Vice Moderate Others:

9. Tutor: Doctor Tutor Master Tutor No

10. Research Direction: Nursing management Nursing Education Medical Nursing Surgical Nursing Critical Nursing Others:

11. Contact: Phone: ; E-mail:

## Part 2: Consensus Questionnaire of *Clinical nurse delirium subtype assessment Knowledge-Attitude-Practice status questionnaire*

Questionnaire introduction:

1. The target participants are clinical nurses who are working in a clinically responsible nursing position in all types hospital, the nurses who are on the sick leave or maternity leave, or internes were excluded.

2. The revised content is marked in **green,** deleted content is marked as **“~~delete~~”** (Order adjustment in the same section has not been marked, order adjustment in the different sections has been marked as **green**)

3. Rationality Score and Importance Score: 5 points = Very rational/ Very important; 4 points = More rational/ More important; 3 points = General rational/ General important; 2 points = Not very rational/ Not very important; 1 point = Strongly not rational; Strongly not important.

4. If you believe that the description of the content is inaccurate or should be deleted, please fill in the "Comments for amendment or deletion" field or indicate "Delete".

5. If you think there are additional content that we have not considered, please add them in the "Suggested additions" blank box, and please judge the importance of the changes and additions in the same way.

6. Please fill in all items.

### Knowledge Section

5 points = Very rational/ Very important; 4 points = More rational/ More important; 3 points = Generally rational/ Generally important; 2 points = Not very rational/ Not very important; 1 point = Strongly not rational; Strongly not important

|  | **Content** | | **Rationality Score** | | | | | **Importance Score** | | | | | | | | **Expert opinions** |
| --- | --- | --- | --- | --- | --- | --- | --- | --- | --- | --- | --- | --- | --- | --- | --- | --- |
|  |  |  | **5** | **4** | **3** | **2** | **1** | **5** | **4** | | **3** | | **2** | | **1** |  |
| Delirium  Part | **1** | True or False: Delirium is an acute reversible mental disorder caused by various diseases.*1   \| ⑴True ⑵False ⑶I don't know \| \| --- \| |  |  |  |  |  |  |  |  | |  | |  | |  |
|  | **2** | Multiple Choice: What are the risks of delirium?*1234  ⑴Increased mortality;  ⑵Prolonged hospitalization;  ⑶Increased hospitalization costs;  ⑷Residual long-term perceptual impairment;  ⑸I don't know |  |  |  |  |  |  |  |  | |  | |  | |  |
|  | 3 | Multiple Choice: Which groups are at high risk for delirium?*1234  ⑴ICU patients; ⑵Post-operative patients; ⑶Elderly patients; ⑷Palliative care patients; ⑸I don't know |  |  |  |  |  |  |  |  | |  | |  | |  |
|  | **4** | \| Multiple Choice: What are the risk factors for delirium?*12345  ⑴Patient factors:**age, whether combined with underlying diseases, etc.;**  ⑵Drug factors: **sedative drugs, analgesic drugs, etc.;**  ⑶Surgical factors: **type of surgery, postoperative pain, etc.;**  ⑷Environmental factors: **lights, machine alarms, etc.;**  ⑸Psychological factors: **anxiety, depression, sense of stress, etc.;**  ⑹I don't know  **（The explanation of each selection has been added）** \| \| --- \| |  |  |  |  |  |  |  |  | |  | |  | |  |
|  | **5** | Multiple Choice: What are the clinical features of delirium?*12345  ⑴Inability to concentrate;  ⑵Disorganized thinking;  ⑶Increased activity;  ⑷Decreased activity;  ⑸Altered state of consciousness;  ⑹I don't know |  |  |  |  |  |  |  |  | |  | |  | |  |
|  | **6** | Multiple Choice: **Which of the following tools are delirium assessment tools?*12345**   \| ⑴DSM-5; ⑵ICD-10; ⑶CAM; ⑷CAM-ICU; ⑸ICDSC; ⑹I don't know \| \| --- \| |  |  |  |  |  |  |  |  | |  | |  | |  |
|  | **7** | Multiple Choice: What are the management measures after the occurrence of delirium?*12345  ⑴Treatment of the cause;  ⑵Early activity;  ⑶Emphasis on sleep management;  ⑷Intensive management;  ⑸**Targeted treatment according to different subtypes of delirium; (Added selection)**  ⑹I don't know |  |  |  |  |  |  |  |  | |  | |  | |  |
|  | **8** | Multiple Choice: Key strategies to prevent and reduce delirium include which?*1234  ⑴Identify and modify risk factors that lead to delirium;  ⑵Early detection of patients at risk for delirium;  ⑶Pay attention to patients' sleep management;  ⑷Help patients at risk for delirium to perform early rehabilitation activities  **(~~Timely subtype assessment of high-risk patients and targeted preventive treatment for different types of delirium~~**  ⑸Take timely restraint measures for patients with delirium;  ⑹I don't know |  |  |  |  |  |  |  |  | |  | |  | |  |
| Delirium Subtypes Part | **9** | **Depart this question in last vision into two questions to clarity the clinical manifestations and adverse outcomes.**  **~~Multiple Choice: What is correct about the following selection of delirium subtypes? *12345~~**  **~~⑴Hypoactive delirium is characterized by emotional poverty, indifference, drowsiness, and decreased reactivity;~~**  **~~⑵Hyperactive delirium is characterized by agitation, anxiety, and attempts to catch extubation;~~**  **~~⑶Mixed delirium shows fluctuations in agitation and quiet symptoms;~~**  **~~⑷Patients with increased activity delirium are relatively more likely to have adverse events such as falls, bed falls, and catheter extraction accidents;~~**  **~~⑸Decreased activity delirium is less likely to be noticed by health care workers and has a more serious impact on patients;~~**  **~~⑹I don’t know~~**  **What is correct about the following clinical manifestations of each subtype of delirium?*123**  ⑴Hyporactive delirium is characterized by emotional poverty, indifference, drowsiness, and decreased reactivity;  ⑵Hyperactive delirium is characterized by agitation, anxiety, and attempts to catch extubation;  ⑶Mixed delirium shows fluctuations in agitation and quiet symptoms;  **~~⑷Patients with hyperactive delirium often have single clinical features with better outcomes;~~**  **~~⑸Hypoactive delirium is more likely to be ignored, so it has severer influence;~~**  ⑷I don’t know |  |  |  |  |  |  |  |  | |  | |  | |  |
|  | **10** | **Multiple Choice: What is correct about the following adverse outcomes for each subtype of delirium?*134**  **⑴Patients with increased activity delirium are relatively more likely to have adverse events such as falls, bed falls, and catheter extraction accidents;**  **⑵Patients with increased activity delirium are less likely to be detected by health care personnel;**  **⑶Patients with decreased activity delirium are more likely to have stress injuries;**  **⑷Decreased activity delirium is less likely to be noticed by health care personnel and has more severe effects on patients;**  **⑸It is not clear and cannot be judged** |  |  |  |  |  |  |  |  | |  | |  | |  |
|  | **11** | Multiple Choice: Which of the following are delirium subtype assessment tools?*234  ⑴ICDSC; ⑵RASS; ⑶DMSS; ⑷MDAS; ⑸I don't know |  |  |  |  |  |  |  |  | |  | |  | |  |
| **Addition** |  |  |  |  |  |  |  |  |  |  | |  | |  | |  |
|  |  |  |  |  |  |  |  |  |  |  | |  | |  | |  |

### Attitude Section

5 points = Very rational/ Very important; 4 points = More rational/ More important; 3 points = Generally rational/ Generally important; 2 points = Not very rational/ Not very important; 1 point = Strongly not rational; Strongly not important

|  | **Content** | **Rationality Score** | | | | | **Importance Score** | | | | | **Expert opinions** |
| --- | --- | --- | --- | --- | --- | --- | --- | --- | --- | --- | --- | --- |
|  |  | **5** | **4** | **3** | **2** | **1** | **5** | **4** | **3** | **2** | **1** |  |
| **1** | Scoring: How important do you think nursing care is in preventing and recovering delirium? |  |  |  |  |  |  |  |  |  |  |  |
| **2** | Scoring: Do you think clinical nurses should undertake the identification of delirium and delirium subtypes? |  |  |  |  |  |  |  |  |  |  |  |
| **3** | Scoring: Do you think clinical nurses should know delirium and delirium subtypes? |  |  |  |  |  |  |  |  |  |  |  |
| **4** | Scoring: Do you think your knowledge of delirium and delirium subtypes can meet clinical needs? |  |  |  |  |  |  |  |  |  |  |  |
| **5** | Scoring: Are you interested in knowledge about delirium and delirium subtypes? |  |  |  |  |  |  |  |  |  |  |  |
| **6** | Scoring: Do clinical nurses need to learn about delirium and its subtypes actively? |  |  |  |  |  |  |  |  |  |  |  |
| **7** | Scoring: Do you think clinical nurses must receive systematic training on knowledge related to delirium and delirium subtypes? |  |  |  |  |  |  |  |  |  |  |  |
| **8** | Scoring: Do you think conducting a delirium subtype assessment in clinical work is necessary? |  |  |  |  |  |  |  |  |  |  |  |
| **9** | Scoring: Do you think it is necessary to develop/introduce delirium subtype assessment tools? |  |  |  |  |  |  |  |  |  |  |  |
| **10** | Scoring: Are you willing to receive training on delirium subtypes? |  |  |  |  |  |  |  |  |  |  |  |
| **11** | Multiple Choice: Which of the following types of delirium have you heard of?  ⑴Hyperactive delirium;  ⑵Hypoactive delirium  ⑶Mixed delirium;  ⑷Quiet delirium;  ⑸Excited delirium;  ⑹Depressed delirium;  ⑺No motor delirium;  ⑻Mixed delirium;  ⑼Other ___________ (please fill in);  ⑽ none of the above have been heard of |  |  |  |  |  |  |  |  |  |  |  |
| **12** | Multiple Choice: What are your requirements for delirium assessment tools?  ⑴Accurate assessment results;  ⑵Reasonable assessment time;  ⑶Reasonable frequency of assessment;  ⑷Clear and easy to understand the text;  ⑸Concise and clear forms;  ⑹Other ___________ (please fill in) |  |  |  |  |  |  |  |  |  |  |  |
| **13** | **Depart this question in last vision into two questions to clarity the individual barriers and organizational barriers.**  **Multiple Choice: At the individual level, what are the current barriers to early delirium recognition for nurses?**  ⑴Insufficient knowledge base of delirium;  ⑵Insufficient mastery of delirium assessment methods;  ⑶Insufficient proficiency in the use of delirium assessment scales;  ⑷Delirium assessment increases workload;  ⑸Nurses are not confident enough in terms of their ability to assess delirium and do not trust the results of their assessment;  ⑹Nurses are busy with clinical work and lack time to conduct delirium assessments;  ⑺Nurses do not cooperate sufficiently with physicians;  ⑻Other _________________(please fill in) |  |  |  |  |  |  |  |  |  |  |  |
| **14** | **Multiple Choice: At the organizational level, what are the current barriers to nurses' early identification of delirium? (Added question)**  ⑴The department/hospital does not have process specifications related to delirium assessment;  ⑵The department/hospital does not conduct training related to delirium assessment;  ⑶The department does not routinely conduct delirium assessments;  ⑷The department does not provide delirium assessment tools;  ⑸The department's human resource allocation is inadequate;  ⑹Other _________________(please fill in) |  |  |  |  |  |  |  |  |  |  |  |
| **15** | **~~（If you score 1 to 3 points, please answer following question)~~ (Change this question into required question and delete the limitation)**  **~~Why do you think it is not necessary to assess delirium subtypes?~~**  **Multiple Choice: In your opinion, what are the barriers to delirium subtype assessment?**  ⑴Delirium assessment work is still immature, and subtype assessment work is not carried out at all;  ⑵There is no significant difference in the clinical manifestations of each delirium subtype;  ⑶There is no significant difference in the management measures of each delirium subtype;  ⑷There is no significant difference in the prognostic impact of each delirium subtype;  **~~⑸The department's human resource allocation is inadequate;~~**  **~~⑹The department does not provide delirium assessment tools;~~**  **~~⑺Nurses have little communication with patients~~**  **~~⑻Nurses do not cooperate sufficiently with physicians;~~**  **⑸Nurses are busy with clinical work, and delirium subtype assessment will increase the workload of nurses; (Added selection)**  **⑹Nurses do not have enough knowledge of delirium subtypes and assessment methods;(Added selection)**  **⑺Nurses lack objective delirium subtype assessment tools;(Added selection)**  **⑻The department/hospital does not focus on this part of the delirium subtype assessment at present and does not request the staff to assess the delirium subtype;(Added selection)**  ⑼Other __________(please fill in) |  |  |  |  |  |  |  |  |  |  |  |
| **16** | Single Choice: How well do you think delirium assessment is done in the section you work in? (If it is convenient, please briefly describe the problems that exist)  ⑴Very well done;  ⑵Basically well done, but still some details are not enough (please describe it: __________optional blank);  **⑶~~Generally done, but still need to improve (please describe it: __________optional blank);~~ (Repeated meaning as last selection)**  ⑷Not well done, still many problems (please describe it: __________optional blank);  **⑸No delirium subtypes assessment work at all** |  |  |  |  |  |  |  |  |  |  |  |
| **Addition** |  |  |  |  |  |  |  |  |  |  |  |  |
|  |  |  |  |  |  |  |  |  |  |  |  |  |

### Practice Section

5 points = Very rational/ Very important; 4 points = More rational/ More important; 3 points = Generally rational/ Generally important; 2 points = Not very rational/ Not very important; 1 point = Strongly not rational; Strongly not important

|  | **Content** | **Rationality Score** | | | | | **Importance Score** | | | | | **Expert opinions** |
| --- | --- | --- | --- | --- | --- | --- | --- | --- | --- | --- | --- | --- |
|  |  | **5** | **4** | **3** | **2** | **1** | **5** | **4** | **3** | **2** | **1** |  |
| **1** | Single Choice: In your daily clinical work, do you assess delirium?  ⑴Always; ⑵Often; ⑶Sometimes; ⑷Sometimes; ⑸Never |  |  |  |  |  |  |  |  |  |  |  |
| **2** | Single Choice: How do you assess and document delirium in your clinical work?  ⑴Assessed by diagnostic scales and recorded;  ⑵Assessed by diagnostic scales but not recorded;  ⑶Assessed by clinical experience only and recorded;  ⑷Assessed by clinical experience only and not recorded;  ⑸ Did not assess delirium |  |  |  |  |  |  |  |  |  |  |  |
| **2.1** | （If you selected (1) or (3) for question 2, please answer following questions)  Single Choice: What do you record about delirium? (⑴ or ⑶ was selected for 3.2)  A. "Patient has delirium of type xxx."  B. "Patient has delirium."  C. "Patient has confusion."  D. "Patient has Behavioral mental abnormalities."  E. Other, please describe _________________ |  |  |  |  |  |  |  |  |  |  |  |
| **2.1.1** | Multiple Choice: Why don't you record this as "patient has delirium"? ( C or D was selected for 2.1)  (1) No delirium diagnostic tool was used for assessment;  (2) Diagnostic tool was used but still not sure if the patient had delirium;  (3) Physician did not make a diagnosis of delirium;  (4) In the nursing records of the department, such patients were recorded as "confusion/abnormal mental behavior";  (5) Other _____ (please fill in) |  |  |  |  |  |  |  |  |  |  |  |
| **2.2** | （If you selected (1) or (2)for question 2, please answer following questions)  **~~Which scales did you use? Please fill in the blank.~~**  **Changed it into single choice question.**  **What is the scale you use most frequently?**  **A.CAM; B.CAM-ICU; C.ICDSD; D.Nu-DESC; E.Other _____ (please fill in)** |  |  |  |  |  |  |  |  |  |  |  |
| **3** | Multiple Choice: How do you usually solve delirium problems when you encounter them in your daily clinical work?  ⑴Discuss with doctors to solve the problem;  ⑵Discuss with other nurses to solve the problem;  ⑶**Ask psychiatrists /psychologist for assistance;**  ⑷ Consult psychiatrists /psychologist;  ⑸ Solve the problem independently;  ⑹Other ___________(please fill in) |  |  |  |  |  |  |  |  |  |  |  |
| **4** | Single Choice: In your daily clinical work, do you assess your patients' type of delirium (delirium subtype)?  ⑴always; ⑵often; ⑶sometimes; ⑷occasionally; ⑸ never |  |  |  |  |  |  |  |  |  |  |  |
| **4.1** | Multiple Choice:（If you selected (3) (4) (5)for question 4, please answer following questions)  Please tell me why you do not assess/are less likely to assess delirium subtypes?  A. Do not know about delirium subtypes;  B. Do not have delirium subtype assessment workers  C. Do not know how to use delirium subtype assessment tools;  D. Do not think the assessment is necessary;  E. Other __________ (please fill in)  （If you selected B, please answer following questions)  Fill in the blanks: I am using scale to assess delirium subtypes. |  |  |  |  |  |  |  |  |  |  |  |
| **5** | **Single Choice: Which In your daily clinical work, which of the following delirium patients are more common?**  **⑴Manic patients: increased speech, restlessness, and uncontrolled behavior;**  **⑵Quiet patients: slower movements, reduced speech, and lowered speaking volume;**  **⑶Mixed patients: alternation of the above two manifestations;**  **⑷I cannot distinguish between the above types of delirium patients.** |  |  |  |  |  |  |  |  |  |  |  |
| **5.1** | Multiple Choice: How would you assess the patient's delirium subtype? **(⑴or ⑵ or ⑶ was selected for question 5)**  A. By clinical experience;  B. By assessing with the help of specific scales;  C. By consulting with colleagues;  D. Other ______ (please fill in) |  |  |  |  |  |  |  |  |  |  |  |
| **5.1.1** | Fill in the blanks: I am using scale to assess delirium subtypes. (B was selected for 5.1) |  |  |  |  |  |  |  |  |  |  |  |
| **Addition** |  |  |  |  |  |  |  |  |  |  |  |  |
|  |  |  |  |  |  |  |  |  |  |  |  |  |

### Knowledge Source Section

5 points = Very rational/ Very important; 4 points = More rational/ More important; 3 points = Generally rational/ Generally important; 2 points = Not very rational/ Not very important; 1 point = Strongly not rational; Strongly not important

|  | **Content** | **Rationality Score** | | | | | **Importance Score** | | | | | **Expert opinions** |
| --- | --- | --- | --- | --- | --- | --- | --- | --- | --- | --- | --- | --- |
|  |  | **5** | **4** | **3** | **2** | **1** | **5** | **4** | **3** | **2** | **1** |  |
| **1** | Scoring: Does the knowledge you learned in school about delirium meet the needs of your current clinical work? |  |  |  |  |  |  |  |  |  |  |  |
| **2** | **Single Choice: Have you ever participated in delirium-related knowledge training?**  **⑴Yes; ⑵No**  **~~Single Choice: Have you ever participated in delirium-related knowledge training?~~**  **~~⑴More than 10 times training( including 10); ⑵5 to 9 times training; ⑶2 to 4 times training; ⑷only once; ⑸no training~~** |  |  |  |  |  |  |  |  |  |  |  |
| **2.1** | **(⑴ was selected for question 2)**  **Single Choice: If you have participated in the training, at the end of the training, did you pass the training assessment? (New question)**  **A. all passed;**  **B. can pass about 80% or more of the assessment;**  **C. can pass about 50%-80% of the assessment;**  **D. can pass about 30%-50% of the assessment;**  **E. can only pass about 30% of the assessment;**  **F. no assessment session set** |  |  |  |  |  |  |  |  |  |  |  |
| **2.2** | **(⑴ was selected for question 2)**  Multiple Choice: Which of the following categories/does the knowledge training you attended belong to?  A. Hospital level physician lectures (led by physicians/medical department, etc.);  B. Hospital level nurse lectures (led by nurses or nursing department, etc.);  C. Department level physician lectures (led by physician teaching team leader/director, etc.);  D. Department level nurse lectures (led by nurse teaching team leader/nurse manager, etc.);  E. Outbound training and learning;  F. Participation in academic conferences;  H. Personal initiative to learn relevant knowledge.  I. Other ________(please fill in) |  |  |  |  |  |  |  |  |  |  |  |
| **3** | Multiple Choice: Which of the following sources your knowledge of delirium and delirium subtypes comes primarily from ?  ⑴ Study at school;  ⑵ Academic conferences and lectures;  ⑶ Relevant study classes;  ⑷ Self-study (due to personal interest or work needs);  ⑸ Work experience accumulation;  ⑹ Exchange among colleagues;  ⑺ Relevant media reports;  ⑻ Consult relevant experts;  ⑼ Brochures and publicity wall posters;  ⑽ Others _______________(please fill in) |  |  |  |  |  |  |  |  |  |  |  |
| **4** | Multiple Choice: In what ways would you most like to enhance your knowledge about delirium and delirium subtypes?  ⑴ Study at school;  ⑵ Academic conferences and lectures;  ⑶ Relevant study classes;  ⑷ Self-study (due to personal interest or work needs);  ⑸ Work experience accumulation;  ⑹ Exchange among colleagues;  ⑺ Relevant media reports;  ⑻ Consult relevant experts;  ⑼ Brochures and publicity wall posters;  ⑽ Others _______________(please fill in) |  |  |  |  |  |  |  |  |  |  |  |
| **5** | Multiple Choice: What are you most looking forward to learning about delirium and delirium subtypes? (Select up to 5 items)  ⑴ Definition of delirium;  ⑵ Monitoring and diagnosis of delirium;  ⑶ Risk factors and etiology of delirium;  **~~⑷ Pathophysiology of delirium;~~**  ⑸ Definition and clinical manifestations of delirium subtypes;  ⑹ Assessment methods and assessment tools of delirium subtypes;  ⑺ Nursing measures and nursing priorities of delirium subtypes;  ⑻ Other ______________(please fill in) |  |  |  |  |  |  |  |  |  |  |  |
| **Addition** |  |  |  |  |  |  |  |  |  |  |  |  |
|  |  |  |  |  |  |  |  |  |  |  |  |  |

Please select the basis for your judgement, level of influence and familiarity with the above entry “√” the appropriate column

| Basis of judgement | Level of influence | | | | |
| --- | --- | --- | --- | --- | --- |
|  | High | middle | | Low | |
| Theoretical analysis |  |  | |  | |
| Practical experience |  |  | |  | |
| Literature reading |  |  | |  | |
| Intuitive feeling |  |  | |  | |
| How familiar are you with the content of this survey | | | | | |
| Degree of familiarity | Extremely familiar | Very familiar | Generally familiar | Slightly familiar | Unfamiliar |
|  |  |  |  |  |  |

End of form. Thank you again for your support and help with this subject.

I wish you a happy life. Good luck with your work.
