# Supplementary material for: Development and validation of nurse’s assessment ability questionnaire in delirium subtypes: Based on Delphi expert consensus
Source: PLoS One. 2024 Jan 23;19(1):e0297063. doi: 10.1371/journal.pone.0297063 (PMC10805299; doi:10.1371/journal.pone.0297063)
Supplement: S5 File — (DOCX) [file pone.0297063.s005.docx]

| 维度1:知识部分 | | | |
| --- | --- | --- | --- |
|  | 1.1 | 判断：谵妄是指由各种疾病引起的急性可逆性精神障碍。 | |
|  | 1.2 | 多选：谵妄的危害包括哪些？  ①死亡率上升；②住院时间延长；③住院费用增加；④遗留长期的知觉障碍；不了解 | |
|  | 1.3 | 多选：谵妄的高危人群包括哪些？  ①ICU患者；②术后患者；③老年患者；④姑息治疗患者；不了解 | |
|  | 1.4 | 多选：谵妄的危险因素包括哪些？  ①患者因素：年龄、是否合并基础疾病等；②药物因素：镇静药物、镇痛药物等；③手术因素：手术类型、术后疼痛等；④环境因素：灯光、机器报警声等；⑤心理因素：焦虑、抑郁、压力感等；⑥护理因素：约束、治疗操作等；⑦不了解 | |
|  | 1.5 | 多选：谵妄的临床特征包括哪些？  ①注意力不能集中；②思维紊乱；③活动增多；④活动减少；⑤意识状态改变；⑥不了解 | |
|  | 1.6 | 多选：下列哪些工具是谵妄评估工具？  ①DSM-5；②ICD-10；③CAM；④CAM-ICU；⑤ICDSC；⑥不了解 | |
|  | 1.7 | 多选：发生谵妄后的处理措施包括哪些？  ①对因治疗；②集束化管理；③早期活动；④重视睡眠管理；⑤根据谵妄的不同亚型，进行针对性处理；④不了解 | |
|  | 1.8 | 多选：预防和减少谵妄的关键策略包括哪些？  ①确定和改变导致谵妄的危险因素；②及早发现谵妄高危患者；③重视患者的睡眠管理；④帮助谵妄高危患者进行早期康复活动；⑤及时对谵妄患者采取约束措施；⑥不了解 | |
|  | 1.9 | 多选：下列关于谵妄各亚型的临床表现，正确的是？  ①活动减少型谵妄是以感情贫乏、感情淡漠、嗜睡和反应性降低为特征；②活动增多型谵妄是以躁动、焦虑并试图拔管为特征；③混合型谵妄表现为躁动与安静症状的波动；④不了解 | |
|  | 1.10 | 多选：下列关于谵妄各亚型的不良结局，正确的是？  ①活动增多型谵妄患者相对来说更易发生跌倒、坠床、导管拔出意外等不良事件；②活动增多型谵妄患者更不容易被医护人员察觉；③活动减少型谵妄患者更易发生压力性损伤；④活动减少型谵妄不易引起医护人员注意，对患者影响更为严重；⑤不清楚，无法判断 | |
|  | 1.11 | 多选：下列哪些为谵妄亚型评估工具？  ①ICDSC；②RASS；③DMSS；④MDAS；⑤不了解 | |
| 维度2：态度部分 | | | |
|  | 2.1 | 打分：您认为护理工作对谵妄的预防及恢复有多重要？ | |
|  | 2.2 | 打分：您认为临床护士是否应该承担谵妄及谵妄亚型的识别工作？ | |
|  | 2.3 | 打分：您认为临床护士是否应该掌握谵妄及谵妄亚型的相关知识？ | |
|  | 2.4 | 打分：您认为自身谵妄及谵妄亚型知识是否能够满足临床需要？ | |
|  | 2.5 | 打分：您是否对谵妄及谵妄亚型的相关知识感兴趣？ | |
|  | 2.6 | 打分：您认为临床护士是否有必要主动学习谵妄及谵妄亚型相关知识？ | |
|  | 2.7 | 打分：您认为临床护士有必要接受谵妄及谵妄亚型相关知识系统培训吗？ | |
|  | 2.8 | 打分：在临床工作中，您认为是否有必要进行谵妄亚型评估工作？ | |
|  | 2.9 | 打分：您认为有必要开发/引进谵妄亚型评估工具吗？ | |
|  | 2.10 | 打分：您愿意接受谵妄亚型的相关知识培训吗？ | |
|  | 2.11 | 多选：以下类型的谵妄您曾听说过哪些？  ①活动增多型谵妄；②活动减少型谵；③狂躁型谵妄；④安静型谵妄；⑤兴奋型谵妄；⑥抑郁型谵妄；⑦无活动型谵妄；混⑧合型谵妄；⑨其他 ___________（请填写）；⑩以上都没有听说过 | |
|  | 2.12 | 多选：您对谵妄评估工具的要求有哪些？  ①评估结果准确；②评估用时合理；③评估频次合理；④文字清晰易懂；⑤表格简洁明了；⑥其他 | |
|  | 2.13 | 多选：您认为，在个人层面，目前影响护士早期识别谵妄的障碍因素有哪些？  ①谵妄知识储备不足；②谵妄评估方法掌握不足；③谵妄评估量表使用不够熟练；④谵妄评估会增加工作量；⑤护士对自己谵妄评估的能力方面不够自信，不相信自己评估的结果；⑥护士临床工作繁忙，缺乏时间进行谵妄评估；⑦护士与医生合作不够充分；⑧其他 _________________ | |
|  | 2.14 | 多选：您认为，在组织层面，目前影响护士早期识别谵妄的障碍因素有哪些？  ①科室/医院没有谵妄评估的相关流程规范；②科室/医院没有开展谵妄评估的相关培训；③科室没有常规进行谵妄评估；④科室没有提供谵妄评估工具；⑤科室人力资源配置不足；⑥其他 _________________ | |
|  | 2.15 | 多选：您认为，谵妄亚型评估的障碍因素有哪些？  ①各个谵妄亚型的临床表现没有太大差别；②各个谵妄亚型的处理措施没有太大差别；③各个谵妄亚型的对预后的影响没有太大差别；④护士的临床工作繁忙，谵妄亚型评估会增加护士的工作量；⑤护士对谵妄亚型知识和评估方法掌握不足；⑥护士缺乏客观的谵妄亚型评估工具；⑦谵妄评估工作尚且不成熟，亚型评估工作完全没有开展；⑧科室/医院现目前没有关注谵妄亚型的评估这部分内容；⑨其他__________ | |
|  | 2.16 | 单选：您认为在您工作的科室中谵妄评估工作做得怎么样？（如果方便，请您简要描述存在的问题）  A做得非常完善；B做得基本完善，仍有些细节不足__________；C做得不太好，仍有很多问题 ___________；D完全没有开展谵妄评估工作 | |
| 维度3：行为部分 | | | |
|  | 3.1 | 单选：在日常的临床工作中，您是否评估了谵妄？  A.总是；B.经常；C.有时；D.偶尔；E.从不 | |
|  | 3.2 | 单选：您在临床工作中是如何评估和记录谵妄的？  A.通过诊断量表进行评估，并记录；B.通过诊断量表进行评估，但未记录；C.仅靠临床经验进行评估，并记录；D.仅靠临床经验进行评估，并未记录；E.并未对谵妄进行评估 | |
|  |  | 3.2.1 单选：请问关于谵妄您记录的内容是什么？（3.2选择A或C）  A.“患者存在xxx型谵妄” B.“患者存在谵妄” C.“患者存在意识模糊” D.“患者存在精神行为异常” E.其他，请您描述 _________________ | |
|  |  | | 3.2.1.1 多选：请问您为什么不记录为“患者存在谵妄”？ （3.2.1选择 C或D）  ①未使用谵妄诊断工具进行评估；②使用了诊断工具但仍不确定患者是否存在谵妄；③医生没有下谵妄诊断；④在科室的护理记录中此类患者均记录“意识模糊/精神行为异常”⑤其他_____（请填写） |
|  |  | 3.2.2 单选：请问您使用频率最高的量表是什么？（3.2选择A或B）  A.CAM（意识模糊评估量表）；B.CAM-ICU（ICU意识模糊评估量表）；C：ICDSD（ICU谵妄筛查量表）；D.Nu-DESC（护理谵妄筛查量表）；E其他 _________ | |
|  | 3.3 | 多选：在日常的临床工作中，您遇到谵妄问题时一般怎么进行解决？  ①和医生商量解决；②和其他护士商量解决；③请精神科/心理科医生解决；④咨询精神科/心理科医生；⑤独立解决；⑥其他 _________________ | |
|  | 3.4 | 单选：在日常的临床工作中，请问您是否评估了患者谵妄的类型（谵妄亚型）？  A.总是；B.经常；C.有时；D.偶尔；E.从不 | |
|  |  | 3.4.1 多选：请问您为何不评估/较少评估谵妄亚型？（3.4选择C或D或E）  ①不了解谵妄亚型相关知识；②没有谵妄亚型评估工具；③不会使用谵妄亚型评估工具；④认为评估必要性不大；⑤其他 __________（请填写） | |
|  | 3.5 | 单选：在您日常的临床工作中，请问下列哪类谵妄患者更为多见？  A.狂躁型患者：言语增多、躁动不安、行为失控；B.安静型患者：动作减缓、言语减少、说话音量降低；C.混合型患者：以上两种表现交替存在；D.我无法区分以上几类谵妄患者 | |
|  |  | 3.5.1 多选：请问您如何评估患者的谵妄亚型？（3.5选择A或B或C）  ①通过临床经验评估；②通过借助某些量表评估；③通过与同事商量评估；④其他 ______（请填写） | |
|  |  | | 3.5.1.1 填空：您借助的是 量表来评估谵妄亚型。（3.5.1选择②） |
| 维度4：知识来源部分 | | | |
|  | 4.1 | 打分：您在学校所学的谵妄相关知识能否满足当前临床工作的需要？ | |
|  | 4.2 | 单选：您是否参加过谵妄相关知识培训？  A.参加过；B.从未参加过 | |
|  |  | 4.2.1单选：如果您曾参加过培训，在培训结束后，请问您是否通过了培训考核？（4.2选择A）  A.全部通过；B.能够通过约80%以上的考核；C.能够通过约50%-80%的考核；D.能够通过约30%-50%的考核；E.仅能通过约30%的考核；F.没有设置考核环节 | |
|  |  | 4.2.2多选：您参加的知识培训属于以下哪个/哪些类别？（4.2选择A）  ①医院层面医生讲座（由医生/医务科等牵头开展）；②医院层面护士讲座（由护士/护理部等牵头开展）；③科室层面医生讲座（由医生教学组长/主任等牵头开展）④科室层面护士讲座（由护士教学组长/护士长等牵头开展）；⑤外出培训学习；⑥参加学术会议；⑦个人主动学习相关知识；⑧其他 ________ | |
|  | 4.3 | 多选：您的谵妄及谵妄亚型的知识主要来源于下列哪些途径？ | |
|  | 4.4 | 多选：您最希望通过哪些途径加强自己的谵妄及谵妄亚型相关知识？ | |
|  |  | 4.3&4.4选项：①在校学习；②学术会议、讲座；③相关学习班；④自学（因个人兴趣或工作需要）；⑤工作经验积累；⑥同事之间交流；⑦相关媒体报道；⑧请教相关专家；⑨宣传册及宣传墙报；⑩其他_______________ | |
|  | 4.5 | 您现在最期望学习的谵妄及谵妄亚型的内容有哪些？（最多选择5项）  ①谵妄的定义；②谵妄的监测及诊断；③谵妄的危险因素及病因；④谵妄的预防及处理；⑤谵妄亚型的定义及临床表现；⑥谵妄亚型的评估方式及评估工具；⑦谵妄亚型的护理措施及护理重点；⑧其他______________ | |
